# Association of Antenatal Diet and Physical Activity–Based Interventions With Gestational Weight Gain and Pregnancy Outcomes: A Systematic Review and Meta-analysis

**DOI:** 10.1001/jamainternmed.2021.6373

**Published:** 2021-12-20

**Authors:** Helena J. Teede, Cate Bailey, Lisa J. Moran, Mahnaz Bahri Khomami, Joanne Enticott, Sanjeeva Ranasinha, Ewelina Rogozińska, Helen Skouteris, Jacqueline A. Boyle, Shakila Thangaratinam, Cheryce L. Harrison

**Affiliations:** 1 Monash Centre for Health Research and Implementation, School of Public Health and Preventive Medicine, Monash University, Melbourne, Victoria, Australia; 2 Endocrinology and Diabetes Units, Monash Health, Melbourne, Victoria, Australia; 3 Warwick Business School, Warwick University, Coventry, United Kingdom; 4 Meta-Analysis Group, Institute of Clinical Trials and Methodology, Medical Research Council Clinical Trials Unit at University College London, London, United Kingdom; 5 Monash Women’s, Monash Health, Melbourne, Victoria, Australia; 6 Institute of Metabolism and Systems Research, University of Birmingham, Birmingham, United Kingdom; 7 Birmingham Women’s and Children’s National Health Service Foundation Trust, Birmingham, United Kingdom

## Abstract

**Importance:**

Excessive gestational weight gain (GWG) is common and associated with adverse pregnancy outcomes. Antenatal lifestyle interventions limit GWG; yet benefits of different intervention types and specific maternal and neonatal outcomes are unclear.

**Objective:**

To evaluate the association of different types of diet and physical activity–based antenatal lifestyle interventions with GWG and maternal and neonatal outcomes.

**Data Sources:**

A 2-stage systematic literature search of MEDLINE, Embase, Cochrane Database of Systematic Reviews, Database of Abstracts of Reviews of Effects, Cochrane Central Register of Controlled Trials, and Health Technology Assessment Database was conducted from February 1, 2017, to May 31, 2020. Search results from the present study were integrated with those from a previous systematic review from 1990 to February 2017.

**Study Selection:**

Randomized trials reporting GWG and maternal and neonatal outcomes.

**Data Extraction and Synthesis:**

Data were extracted for random-effects meta-analyses to calculate the summary effect estimates and 95% CIs.

**Main Outcomes and Measures:**

Outcomes were clinically prioritized, with mean GWG as the primary outcome. Secondary outcomes included gestational diabetes, hypertensive disorders of pregnancy, cesarean section, preterm delivery, large or small for gestational age neonates, neonatal intensive care unit admission, or fetal death.

**Results:**

A total of 117 randomized clinical trials of antenatal lifestyle interventions (involving 34 546 women) were included. Overall lifestyle intervention was associated with reduced GWG (−1.15 kg; 95% CI, −1.40 to −0.91), risk of gestational diabetes (odds ratio [OR], 0.79; 95% CI, 0.70-0.89), and total adverse maternal outcomes (OR, 0.89; 95% CI, 0.84-0.94) vs routine care. Compared with routine care, diet was associated with less GWG (−2.63 kg; 95% CI, −3.87 to −1.40) than physical activity (−1.04 kg; 95% CI, −1.33 to −0.74) or mixed interventions (eg, unstructured lifestyle support, written information with weight monitoring, or behavioral support alone) (−0.74 kg; 95% CI, −1.06 to −0.43). Diet was associated with reduced risk of gestational diabetes (OR, 0.61; 95% CI, 0.45-0.82), preterm delivery (OR, 0.43; 95% CI, 0.22-0.84), large for gestational age neonate (OR, 0.19; 95% CI, 0.08-0.47), neonatal intensive care admission (OR, 0.68; 95% CI, 0.48-0.95), and total adverse maternal (OR, 0.75; 95% CI, 0.61-0.92) and neonatal outcomes (OR, 0.44; 95% CI, 0.26-0.72). Physical activity was associated with reduced GWG and reduced risk of gestational diabetes (OR, 0.60; 95% CI, 0.47-0.75), hypertensive disorders (OR, 0.66; 95% CI, 0.48-0.90), cesarean section (OR, 0.85; 95% CI, 0.75-0.95), and total adverse maternal outcomes (OR, 0.78; 95% CI, 0.71-0.86). Diet with physical activity was associated with reduced GWG (−1.35 kg; 95% CI, −1.95 to −0.75) and reduced risk of gestational diabetes (OR, 0.72; 95% CI, 0.54-0.96) and total adverse maternal outcomes (OR, 0.81; 95% CI, 0.69-0.95). Mixed interventions were associated with reduced GWG only.

**Conclusions and Relevance:**

This systematic review and meta-analysis found level 1 evidence that antenatal structured diet and physical activity–based lifestyle interventions were associated with reduced GWG and lower risk of adverse maternal and neonatal outcomes. The findings support the implementation of such interventions in routine antenatal care and policy around the world.

## Key Points

**Question:**

Do different types of antenatal diet and physical activity interventions reduce gestational weight gain and improve maternal and neonatal outcomes?

**Findings:**

In this systematic review and meta-analysis of 117 randomized clinical trials (involving 34 546 pregnancies), antenatal diet and physical activity–based lifestyle interventions were associated with less gestational weight gain. Structured diet, physical activity, and diet with physical activity were all associated with improved maternal outcomes; only diet was associated with improved neonatal outcomes.

**Meaning:**

The findings support the implementation of structured diet and physical activity–based interventions in antenatal care programs and policies across the world.

## Teaser

This systematic review and meta-analysis reports the implications of various lifestyle interventions for gestational weight gain and maternal and neonatal outcomes.

## Introduction

With an obesogenic environment, unhealthy lifestyle, and accelerating weight gain, obesity is now the most common medical condition in the world, projected to affect 21% of women globally by 2025.^1^ In the US, obesity prevalence is higher, affecting 25% of women who become pregnant.^2^ Preconception and pregnancy are priority life stages for healthy lifestyles and obesity prevention,^3,4^ with excess weight being associated with adverse pregnancy outcomes, long-term noncommunicable disease in women, and epigenetic consequences across generations.^4,5,6^ In meta-analyses of more than 1.3 million pregnancies worldwide, gestational weight gain (GWG) that exceeds international recommendations affected approximately half of pregnancies^6,7^ and was an independent risk factor in adverse maternal and neonatal pregnancy outcomes.^5,6,8^ The US Preventive Services Task Force has prioritized antenatal lifestyle interventions to limit excessive GWG,^9^ yet the optimal intervention type and specific associations with maternal and neonatal outcomes remain unclear.

Previous individual patient data meta-analyses across 36 randomized clinical trials (RCTs) in 12 526 women noted that antenatal lifestyle interventions were associated with reduced GWG by 0.7 kg (95% CI, −0.92 to −0.48 kg) and reduced cesarean section by 9% (odds ratio [OR], 0.91; 95% CI, 0.83- 0.99).^10^ In another systematic review of 68 studies and 25 789 participants, antenatal lifestyle interventions were associated with a decrease in GWG and emergency cesarean sections as well as improved neonatal outcomes.^9^ Interventions were broadly classified into active or counseling interventions, with statistical heterogeneity in pooled analyses associated with variability in components.^9^ Further insights into different intervention types are now needed.^7^

We aimed to evaluate the association of different types of diet and physical activity–based antenatal lifestyle interventions with GWG and maternal and neonatal outcomes. We classified the interventions into structured diet, structured physical activity, and diet with physical activity with at least 1 structured component. Other interventions were captured as mixed, which predominantly included unstructured lifestyle support, written information with weight monitoring, or behavioral support alone. We focused on clinically prioritized maternal and neonatal outcomes and aimed to generate level 1 evidence to underpin health economic analysis, public health guidelines, and implementation into policy and practice.^11^

## Methods

### Search Strategy and Selection Criteria

For this systematic review and meta-analysis, a 2-stage search of the literature was conducted across MEDLINE, Embase, Cochrane Database of Systematic Reviews, Database of Abstracts of Reviews of Effects, Cochrane Central Register of Controlled Trials, and Health Technology Assessment Database between February 1, 2017, and May 31, 2020. Search results from the present study were integrated with those from a previous systematic review, which was performed from January 1990 to February 2017.^10^ Search terms and outcomes were clinically prioritized and have been previously published.^10,12^ Bibliographies of included studies were also reviewed to identify additional studies. There were no language restrictions. We followed the Preferred Reporting Items for Systematic Reviews and Meta-analyses (PRISMA) reporting guideline. This study has been registered in PROSPERO (CRD42013003804).

Search methods from the 2017 systematic review were applied, guided by consistent authorship and the registered protocol.^10^ Updated search results to May 2020 were screened by title and abstract by 2 independent investigators (including E.R., for the 2017-2018 search; C.B. and M.B.K., for the 2017-2020 search) who screened the full text of eligible studies. Discrepancies were resolved by a third reviewer (H.J.T.). We included antenatal RCTs of interventions based on diet and/or physical activity, with or without behavioral modification. We excluded studies that targeted maternal conditions that are known to affect GWG (eg, gestational diabetes), involved animals, evaluated nonlifestyle-based interventions (GWG-monitoring RCTs alone), reported only nonclinical outcomes, included weight-reducing drugs or surgical interventions, or were published before 1990.^12^ The comparators were routine antenatal care with outcomes that were clinically prioritized.^13^

Two of us (L.J.M., a dietitian, and C.L.H., an exercise physiologist) independently classified the interventions, and discrepancies were resolved by a third reviewer (H.J.T.). The classifications were structured diet, structured physical activity, diet with physical activity, and mixed interventions. Structured diet interventions used dietary targets, either self-directed or facilitator-led (by researcher, instructor, trainer, or dietitian), with or without monitoring (logs, recalls, or diaries) or supply of food. Structured physical activity interventions involved specified physical activity programs conducted in controlled conditions (research facility, gym, or class) or a few physical activity interventions that were self-led (activity targets and equipment provided). We also extended the initial protocol by separating diet with physical activity interventions (with at least 1 having a structured component) from the original mixed interventions to create 4 intervention types. Residual mixed interventions did not meet the inclusion criteria for structured interventions and focused on unstructured lifestyle support, written information with weight monitoring, or behavioral support alone, or they inadequately described the structured diet and physical activity components. Behavioral strategies were heterogeneously applied across all intervention types, preventing a separate analysis.

The primary outcome was mean GWG. Secondary outcomes included adverse maternal (gestational diabetes; hypertensive disorders of pregnancy encompassing pregnancy-induced hypertension and preeclampsia; any cesarean section; and preterm delivery) and neonatal (large for gestational age [LGA] or small for gestational age [SGA] neonates; newborn admission to a neonatal intensive care unit [NICU]; or fetal death, encompassing intrauterine fetal death and stillbirth) outcomes. Composite outcomes could not be generated from aggregate data; hence, we evaluated total adverse outcomes. All outcomes were clinically prioritized in a previously published Delphi survey.^13^

We accepted the primary clinical trial^10^ definitions and reporting of GWG, gestational diabetes, hypertensive disorders of pregnancy, cesarean section, fetal death, and admission to NICU. We defined preterm delivery as birth before 37 weeks’ gestation, SGA as birth weight lower than the 10th percentile for gestational age, and LGA as birth weight at or more than the 90th percentile for the gestational age, adjusted for the mother’s body mass index, parity, and gestational age at delivery. When these definitions varied, we excluded the outcomes for that particular variable.

### Statistical Analysis

Two researchers (including C.B.) assessed risk of bias using the Cochrane Risk of Bias Tool, version 1.0.^14^ Discrepancies were resolved by consensus with a third reviewer (C.L.H.). Methodological quality of 6 study domains was assessed using the *Cochrane Handbook for Systematic Reviews of Interventions* templates: randomization, allocation concealment, blinding of participants, blinding of outcome assessment, incomplete outcome data, and selective outcome reporting.^14^ The nature of lifestyle interventions made blinding of participants generally not feasible given that selective reporting was rarely documented.^15^ Hence, we calculated risk of bias according to 4 study domains: randomization, allocation concealment, blinding of outcome assessment, and incomplete outcome data. We considered a study at high risk of bias if it scored as such in at least 1 domain. For low risk of bias, all domains had to be scored as low risk.

We assessed the association of interventions with primary and secondary outcomes by calculating the mean differences in continuous ratios and ORs for dichotomous outcomes using the intention-to-treat principle. Random-effects meta-analysis was used to calculate the summary effect estimates and 95% CIs for the intervention effects; the DerSimonian and Laird method was applied using the *metan* Stata command.^16^ Heterogeneity was assessed with the *I*^2^ statistic, and *I*^2^ greater than 50% indicated substantial heterogeneity.

We evaluated the differential implications of interventions by performing a subgroup meta-analysis by intervention type (diet, physical activity, diet with physical activity, and mixed). In addition, we conducted sensitivity analyses to bring the structured interventions (diet, physical activity, and diet with physical activity) together, repeating the meta-analyses that omitted mixed interventions. We analyzed primary outcomes for studies with a low or high risk of bias. When 10 or more studies were available, publication bias was assessed using Egger test plots. Statistical significance was defined as a 2-sided *P* < .05. All statistical analyses were performed using Stata, version 16 (StataCorp LLC).

## Results

**Figure 1.**
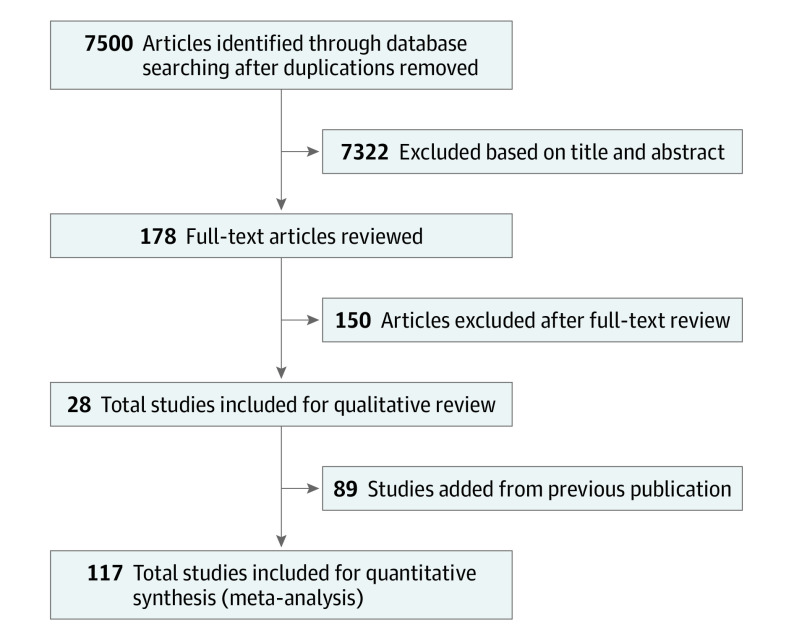
PRISMA Diagram of the Systematic Search

The search identified 7500 studies, of which 178 were retained for full-text review and 28 were included in the present analysis.^17,18,19,20,21,22,23,24,25,26,27,28,29,30,31,32,33,34,35,36,37,38,39,40,41,42,43,44^ In the 2017 systematic review, 103 studies were identified, of which 89 were eligible.^10,12^ The 28 new and 89 previous articles were combined for a total of 117 studies for the present meta-analysis.^10,12,17,18,19,20,21,22,23,24,25,26,27,28,29,30,31,32,33,34,35,36,37,38,39,40,41,42,43,44^ The PRISMA diagram is presented in Figure 1.

This sample consisted of RCTs (which involved 34 546 women) that examined diet (n = 14), physical activity (n = 53), diet with physical activity (n = 19), and mixed interventions (n = 31) (eTable in the Supplement). Forty-four studies were from Europe, 29 were from North America, 13 were from Australia or New Zealand, 9 were from the United Kingdom, 9 were from South America, 7 were from Asia (China, India, or Taiwan), and 6 were from the Middle East (Iran and Egypt). The studies reported on GWG (n = 99), gestational diabetes (n = 67), hypertensive disorders of pregnancy (n = 53), preterm delivery (n = 52), cesarean section (n = 76), fetal death (n = 12), SGA (n = 24) or LGA (n = 28) neonates, and admission to NICU (n = 17).

**Figure 2.**
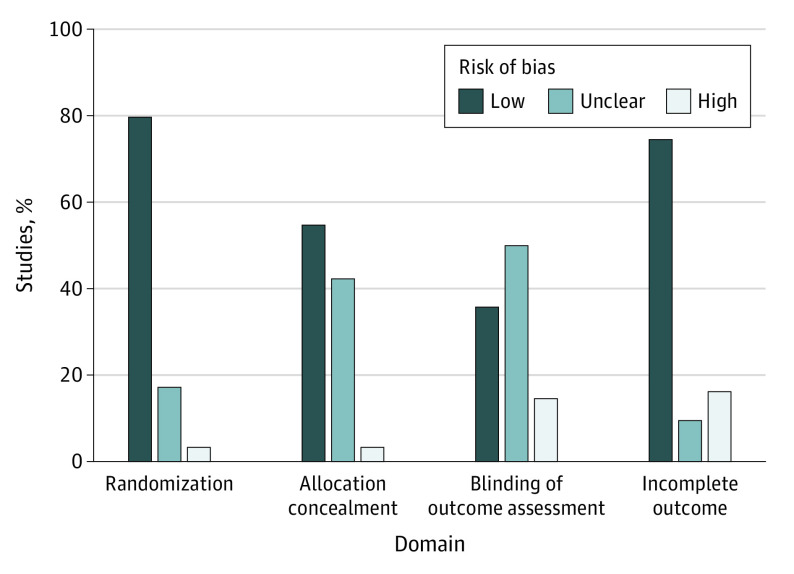
Assessment of Risk of Bias in 4 Domains

Assessment of quality using all 6 study domains (randomization, allocation concealment, blinding of participants, blinding of outcome assessment, incomplete outcome data, and selective outcome reporting) showed that 73 studies (62.4%) had a high risk of bias and 44 (37.6%) had an unclear risk of bias. When evaluating the 4 study domains that were appropriate for lifestyle interventions (randomization, allocation concealment, blinding of outcome assessment, and incomplete outcome data), few studies had a high risk of bias: 22 (18.8%) had a low, 59 (50.4%) had an unclear, and 36 (30.8%) had a high risk of bias (Figure 2).

Visual inspection of the funnel plot for GWG suggested a possible bias against small studies that favored better intervention group outcomes, which was confirmed by Egger test (−9.51; 95% CI, −12.98 to −6.03; *P* < .001) (eFigure 1A in the Supplement). Funnel plots for maternal and neonatal outcomes were largely symmetrical, suggesting a low risk of publication bias, which was supported by Egger test (eFigure 1B in the Supplement).

The GWG results are presented in Table 1 and in a forest plot (eFigure 2 in the Supplement). Overall lifestyle intervention was associated with reduced GWG compared with routine care (−1.15 kg; 95% CI, −1.40 to −0.91; *I*^2^ = 85.3%; 29 247 women). Diet (−2.63 kg; 95% CI, −3.87 to −1.40; *I*^2^ = 94.2%; 4928 women), diet with physical activity (−1.35 kg; 95% CI, −1.95 to −0.75; *I*^2^ = 53.6%; 2942 women), physical activity (−1.04 kg; 95% CI, −1.33 to −0.74; *I*^2^ = 56.2%; 8714 women), and mixed interventions (−0.74 kg; 95% CI, −1.06 to −0.43; *I*^2^ = 70.1%; 12 663 women) were associated with reduced GWG. Diet appeared to have greater implications for weight, with 95% CIs that did not overlap those of physical activity and mixed interventions but were inclusive of that for diet with physical activity. Sensitivity analysis showed that, when analyzed together, structured diet and physical activity interventions (excluding mixed interventions) were associated with reduced GWG of −1.31 kg (95% CI, −1.64 to −0.99; *I*^2^ = 79.0%; 16 584 women), compared with routine care. Analysis by risk of bias showed a mean difference in GWG of −1.23 kg (95% CI, −1.75 to −0.70) for studies with a high risk of bias and −1.13 kg (95% CI, −1.63 to −0.63) for studies with a low risk of bias, with overlapping 95% CIs and no clear differences between the risk of bias study groups.

**Table 1.** Diet and Physical Activity–Based Lifestyle Interventions and Associations With Gestational Weight Gain (GWG)

Intervention	No.		Intervention		Routine care		% Difference^a^	GWG (95% CI), kg	*I*^2^, %
	Studies	Women	Mean weight (SD), kg	Total No.	Mean weight (SD), kg	Total No.			
Overall	99	29 247	0.7 (3.0)	14 861	11.9 (2.9)	14 386	9.7	−1.15 (−1.40 to −0.91)	85.3
Diet	13	4928	8.9 (2.5)	2447	11.6 (3.1)	2481	22.7	−2.63 (−3.87 to −1.40)	94.2
Physical activity	42	8714	11.1 (3.2)	4229	11.9 (3.1)	4485	8.7	−1.04 (−1.33 to −0.74)	56.2
Diet with physical activity	16	2942	10.2 (2.9)	1506	11.6 (2.5)	1436	11.6	−1.35 (−1.95 to −0.75)	53.6
Mixed	28	12 663	11.0 (2.9)	6679	12.0 (2.9)	5984	6.2	−0.74 (−1.06 to −0.43)	70.1

^a^ Lower mean GWG in the intervention group compared with the control group.

For maternal outcomes, overall interventions were associated with reduced risk of gestational diabetes (OR, 0.79; 95% CI, 0.70-0.89; *I*^2^ = 38.3%; 24 371 women) and total adverse maternal outcomes (OR, 0.89; 95% CI, 0.84-0.94; *I*^2^ = 27.9%) compared with routine care (Tables 2 and 3). Diet interventions were associated with lower risk of gestational diabetes (OR, 0.61; 95% CI, 0.45-0.82; *I*^2^ = 25.7%; 3029 women), preterm delivery (OR, 0.43; 95% CI, 0.22-0.84; *I*^2^ = 47.2%; 3379 women), total adverse maternal outcomes (OR, 0.75; 95% CI, 0.61-0.92; *I*^2^ = 47.2%), and total adverse neonatal outcomes (OR, 0.44; 95% CI, 0.26-0.72; *I*^2^ = 48.4%). Physical activity interventions were associated with lower risk of gestational diabetes (OR, 0.60; 95% CI, 0.47-0.75; *I*^2^ = 21.4%; 7519 women), hypertensive disorders of pregnancy (OR, 0.66; 95% CI, 0.48-0.90; *I^2^* = 23.4%; 5332 women), cesarean section (OR, 0.85; 95% CI, 0.75-0.95; *I^2^* = 0.6%; 7528 women), and total adverse maternal outcomes (OR, 0.78; 95% CI, 0.71-0.86; *I^2^* = 13.1%). Diet with physical activity interventions was associated with reduced risk of gestational diabetes (OR, 0.72; 95% CI, 0.54-0.96; *I*^2^ = 29.8%; 3154 women) and total adverse maternal outcomes (OR, 0.81; 95% CI, 0.69-0.95; *I*^2^ = 39.2%) (Tables 2 and 3). Mixed interventions were not associated with maternal or neonatal outcomes. Sensitivity analysis showed that, when analyzed together, structured diet and physical activity interventions (excluding mixed interventions) were associated with reduced risk of gestational diabetes (OR, 0.64; 95% CI, 0.55-0.74; *I*^2^ = 25.1%; 13 702 women), hypertensive disorders of pregnancy (OR, 0.72; 95% CI, 0.58-0.88; *I*^2^ = 32.1%; 10 795 women), and total adverse maternal outcomes (OR, 0.79; 95% CI, 0.73-0.85; *I*^2^ = 28.5%) as well as a pattern of fewer cesarean sections (OR, 0.91; 95% CI, 0.82-1.01; *I*^2^ = 16.4%; 13 138 women).

**Table 2.** Diet and Physical Activity–Based Lifestyle Interventions and Associations With Total Adverse Maternal and Total Adverse Neonatal Outcomes^a^

Intervention	Maternal outcome, OR (95% CI)	*I*^2^, %	Neonatal outcome, OR (95% CI)	*I*^2^, %
Overall	0.89 (0.84-0.94)	27.9	0.94 (0.86-1.04)	17.1
Diet	0.75 (0.61-0.92)	47.2	0.44 (0.26-0.72)	48.4
Physical activity	0.78 (0.71-0.86)	13.1	0.87 (0.67-1.12)	0
Diet with physical activity	0.81 (0.69-0.95)	39.2	0.92 (0.74-1.13)	3.6
Mixed	1.02 (0.97-1.08)	0	1.04 (0.95-1.13)	0

Abbreviation: OR, odds ratio.

^a^ Total adverse maternal outcomes included gestational diabetes, hypertensive disorders of pregnancy, any cesarean section, or preterm delivery. Total adverse neonatal outcomes included large for gestational age or small for gestational age neonates, newborn admission to neonatal intensive care unit, or fetal death.

**Table 3.** Diet and Physical Activity–Based Lifestyle Interventions and Associations With Individual Adverse Maternal and Individual Adverse Neonatal Outcomes

Intervention	No.		Intervention		Routine care		% Difference^a^	OR (95% CI)	*I*^2^, **%**
	Studies	Women	No. of events	Total No. **(**%**)**	No. of events	Total No. **(**%**)**			
**Overall**									
Gestational diabetes	**67**	**24 371**	**1477**	**12 061 (12.2)**	**1732**	**12 310 (14.1)**	**1.8**	**0.79 (0.70-0.89)**	**38.3**
Hypertensive disorders of pregnancy	53	20 811	883	10 363 (8.5)	936	10 448 (9.0)	0.4	0.87 (0.75-1.01)	37.5
Preterm delivery	52	20 083	546	9941 (5.5)	632	10 142 (6.2)	0.7	0.93 (0.80**-**1.07)	11.4
Cesarean section	76	23 333	3053	11 664 (29.4)	3164	11 669 (38.1)	8.7	0.94 (0.88**-**1.01)	14.7
Fetal death	12	7174	20	3558 (0.6)	25	3616 (0.7)	0.1	0.73 (0.40**-**1.32)	0
SGA neonate	24	8747	379	4309 (8.8)	382	4438 (8.6)	−0.2	1.02 (0.86**-**1.12)	6.3
LGA neonate	28	11 432	589	5657 (10.4)	684	5775 (11.8)	1.4	0.83 (0.69**-**1.01)	35.9
NICU admission	17	9613	762	4793 (15.9)	754	4820 15.6)	−0.3	1.02 (0.89**-**1.17)	9.8
**Diet**									
Gestational diabetes	7	3029	183	1490 (12.3)	276	1539 (17.9)	5.7	0.61 (0.45**-**0.82)	25.7
Hypertensive disorders of pregnancy	6	2683	80	1316 (6.1)	97	1367 (7.1)	1.0	0.80 (0.48**-**1.32)	48.8
Preterm delivery	6	3379	65	1658 (3.9)	109	1721 (6.3)	2.4	0.43 (0.22**-**0.84)	47.2
Cesarean section	6	2426	358	1192 (30.0)	354	1234 (28.7)	−1.3	1.07 (0.89**-**1.30)	0
Fetal death	2	1389	1	674 (0.1)	3	715 (0.4)	0.3	0.46 (0.07**-**3.13)	0
SGA neonate	2	974	10	484 (2.1)	28	490 (5.7)	3.6	0.54 (0.06**-**4.64)	83.1
LGA neonate	2	974	6	484 (1.2)	29	490 (5.9)	4.7	0.19 (0.08**-**0.47)	0
NICU admission	3	2092	65	1037 (6.3)	94	1055 (8.9)	2.6	0.68 (0.48**-**0.95)	0
**Physical activity**									
Gestational diabetes	24	7519	219	3603 (6.1)	374	3916 (9.6)	3.5	0.60 (0.47**-**0.75)	21.4
Hypertensive disorders of pregnancy	18	5332	116	2604 (4.5)	182	2728 (6.7)	2.2	0.66 (0.48**-**0.90)	23.4
Preterm delivery	23	6299	154	3057 (5.0)	177	3242 (5.5)	0.4	1.03 (0.81**-**1.29)	0
Cesarean section	34	7528	715	3697 (19.3)	847	3831 (22.1)	2.8	0.85 (0.75**-**0.95)	0.6
Fetal death	2	140	1	66 (1.5)	1	74 (1.4)	−0.2	NA^b^	NA
SGA neonate	9	1265	43	561 (7.7)	65	704 (9.2)	1.6	0.74 (0.48**-**1.15)	0
LGA neonate	9	1236	66	542 (12.2)	85	694 (12.2)	0.1	1.07 (0.69**-**1.68)	16.1
NICU admission	3	997	18	500 (3.6)	25	497 (5.0)	1.4	0.72 (0.39**-**1.35)	0
**Diet with physical activity**									
Gestational diabetes	16	3154	177	1599 (11.1)	215	1555 (13.8)	2.8	0.72 (0.54**-**0.96)	29.8
Hypertensive disorders of pregnancy	13	2780	127	1405 (9.0)	165	1375 (12.0)	3.0	0.74 (0.52**-**1.06)	38.4
Preterm delivery	10	1934	53	973 (5.4)	73	961 (7.6)	2.1	0.75 (0.38**-**1.49)	52.4
Cesarean section	17	3184	346	1622 (21.3)	335	1562 (21.4)	0.1	0.96 (0.75**-**1.23)	38.8
Fetal death	1			0		0	0	NA	NA
SGA neonate	6	1417	78	708 (11.0)	70	709 (9.9)	−1.1	1.15 (0.82**-**1.62)	0
LGA neonate	8	1720	63	867 (7.3)	86	853 (10.1)	2.8	0.71 (0.45**-**1.11)	28.7
NICU admission	4	1040	64	517 (12.4)	68	523 (13.0)	0.6	0.95 (0.66**-**1.38)	0
**Mixed**									
Gestational diabetes	23	10 669	898	5369 (16.7)	867	5300 (16.4)	−0.4	1.03 (0.93**-**1.15)	0
Hypertensive disorders of pregnancy	17	10 016	560	5038 (11.1)	492	4978 (9.9)	−1.2	1.14 (1.00**-**1.30)	0
Preterm delivery	14	8471	274	4253 (6.4)	273	4218 (6.5)	0	1.00 (0.84**-**1.19)	0
Cesarean section	20	10 195	1634	5153 (31.7)	1628	5042 (32.3)	0.6	0.98 (0.89**-**1.08)	11.1
Fetal death	7	5645	18	2818 (0.6)	21	2827 (0.7)	0.1	0.78 (0.40**-**1.51)	0
SGA neonate	9	5091	248	2556 (9.7)	219	2535 (8.6)	−1.1	1.14 (0.94**-**1.38)	0
LGA neonate	11	7502	454	3764 (12.1)	484	3738 (12.9)	0.9	0.92 (0.77**-**1.10)	18.6
NICU admission	7	5484	615	2739 (22.5)	567	2745 (20.7)	−1.8	1.12 (0.98**-**1.29)	0

Abbreviations: LGA, large for gestational age; NA, not applicable (when there was only 1 article in a subcategory, the results were marked NA); NICU, neonatal intensive care unit; SGA, small for gestational age.

^a^ Absolute % difference.

^b^ More than 1 article, but the result was excluded by the meta-analysis process.

For neonatal outcomes, compared with routine care, overall interventions were not associated with the risk of an SGA or LGA neonate, fetal death, NICU admission, or total adverse neonatal outcomes. Diet interventions were associated with a lower risk of NICU admission (OR, 0.68; 95% CI, 0.48-0.95; *I*^2^ = 0%; 2092 women), LGA neonate (OR, 0.19; 95% CI, 0.08-0.47; *I*^2^ = 0%; 974 women), and total adverse neonatal outcomes (OR, 0.44; 95% CI, 0.26-0.72; *I*^2^ = 48.4%) and were not associated with fetal death or SGA neonate (Tables 2 and 3). Other intervention types were not associated with neonatal outcomes. Sensitivity analysis showed that structured diet and physical activity interventions, when analyzed together (excluding mixed interventions), were associated with a lower risk of NICU admission (OR, 0.78; 95% CI, 0.62-0.98; *I*^2^ = 0%; 4129 women) and total adverse neonatal outcomes (OR, 0.78; 95% CI, 0.66-0.92; *I*^2^ = 18.9%).

Data on potential harms of the intervention were limited. However, no association between lifestyle interventions and an SGA neonate was noted.

## Discussion

Excessive GWG is common and associated with increased adverse maternal and neonatal pregnancy outcomes. In this study, we found level 1 evidence^11^ from 117 RCTs, which involved 34 546 women, more than 30 years of research, and 5 continents.^10,12,17,18,19,20,21,22,23,24,25,26,27,28,29,30,31,32,33,34,35,36,37,38,39,40,41,42,43,44^ Compared with routine care, antenatal diet and physical activity–based lifestyle interventions were associated with reduced GWG. Lifestyle interventions were also associated with lower risk of gestational diabetes and total adverse maternal outcomes. Diet interventions seemed to have greater implications for GWG than physical activity alone or mixed interventions, whereas the implications of diet and diet with physical activity for GWG could not be differentiated. Compared with routine care, diet was associated with a reduced risk of gestational diabetes, preterm delivery, total adverse maternal outcomes, LGA neonate, NICU admission, and total adverse neonatal outcomes. Physical activity was associated with a lower risk of gestational diabetes, hypertensive disorders of pregnancy, cesarean section, and total adverse maternal outcomes, whereas diet with physical activity was associated with a reduced risk of gestational diabetes and total adverse maternal outcomes. Mixed interventions were not associated with maternal and neonatal outcomes.

Gestational weight gain that exceeds the recommendations occurs in approximately half of pregnancies and has been associated with adverse maternal and neonatal health outcomes.^5,6,7,45^ Antenatal lifestyle interventions were associated with reduced GWG by 0.7 kg in a previous systematic review and individual patient-level data meta-analysis, with similar efficacy regardless of the mother’s body mass index, age, parity, race and ethnicity, or preexisting medical conditions.^10^ In another systematic review and meta-analysis of 68 studies,^9^ lifestyle interventions were associated with 1.02 kg less GWG with a significant interaction with study intensity.^9^ We found that structured diet and physical activity–based interventions were associated with reduced GWG of 1.15 kg, but 1.13 kg in studies with a low risk of bias.

Regarding intervention types, based on mean GWG and nonoverlapping 95% CIs, diet interventions had greater implications for GWG than physical activity alone or mixed interventions but could not be differentiated from diet with physical activity interventions. The broader weight implications of diet over physical activity alone were consistent with the balance between energy intake and expenditure.^46^ Intake is diet dependent, whereas 60% to 70% of energy expenditure is resting (partly impacted by lean muscle mass) and the residual 30% is used in physical activity. Hence, substantial physical activity is required to achieve an energy deficit and weight loss.^47,48,49^ In the present study, antenatal diet intervention reduced GWG by approximately 23%, likely limiting longer-term obesity and noncommunicable disease risks given the evidence of postpartum weight benefits.^4,9^ Physical activity specifically declines in pregnancy,^50^ with barriers to engagement and improvement.^51^ The present study supports physical activity intervention in pregnancy to improve maternal health outcomes. Because most of these interventions were structured and delivered by trained health professionals alongside routine antenatal care practitioners, we highlight the strong public health argument for implementing structured diet with physical activity lifestyle interventions during pregnancy that are facilitated by trained professionals.

The GWG and public health benefits of pregnancy lifestyle interventions are enhanced by the associated improvement in clinically prioritized maternal outcomes.^13^ Previous meta-analyses inconsistently noted the association of lifestyle intervention with reduced risk of cesarean sections and gestational diabetes^9,10^ but not gestational hypertension. Reported intervention classification has varied, including active interventions with a structured physical element (eg, supervised exercise programs, prescribed exercise or dietary programs, or intensive weight management) or counseling alone. Only active and intensive interventions were associated with reduced risk of gestational hypertension.^9^ We found that lifestyle interventions overall were associated with a reduced risk of gestational diabetes and total adverse maternal outcomes that encompassed gestational diabetes, hypertensive disorders of pregnancy, preterm delivery, and cesarean section. Structured diet interventions were associated with reduced risk of gestational diabetes and preterm delivery, whereas physical activity interventions were associated with reduced risk of gestational diabetes, hypertensive disorders of pregnancy, and cesarean sections. Diet with physical activity interventions were associated with reduced risk of gestational diabetes. Such findings advance existing knowledge, showing broad maternal benefits and differences across intervention types.^9,10^

Lifestyle interventions were also associated with neonatal benefits, which varied across intervention types. The 2017 individual patient data did find neonatal benefits,^10^ but a more recent review found associations with a reduced risk of macrosomia and LGA.^9^ In the present study, diet was associated with reductions in the broadest range of adverse neonatal outcomes, including LGA, NICU admission, and total adverse neonatal outcomes. This current systematic review directly underpinned a cost-effectiveness analysis.^52^ When analyzed together and based on maternity outcomes alone, diet, physical activity, and diet with physical activity interventions appeared to be cost-saving. When NICU costs were incorporated, all except mixed interventions were cost-saving, supporting the implementation of structured lifestyle interventions in pregnancy.

Mixed lifestyle interventions did not include clearly articulated structured diet and physical activity components or encompassed passive lifestyle information or written resources with or without gestational weighing and with or without behavioral strategies. These interventions were associated with limited GWG benefit, with no associations with secondary outcomes. This finding highlights the need for evidence on the most effective intervention components, delivery modes, settings, staffing, and behavioral strategies to inform implementation.^3,53^

Implementation research is underway. A secondary analysis of these 117 interventions is being conducted to identify optimal intervention characteristics via the TIDieR (Template for Intervention Description and Replication) framework.^54,55,56^ Nationally and internationally funded research initiatives, including the Global Alliance of Chronic Disease and Horizon 2020 projects,^3^ are informing the development of an implementation tool kit. This systematic review supports the implementation of structured diet and physical activity interventions by trained staff. It does not support isolated monitoring of GWG and provision of passive lifestyle information by routine antenatal care staff, an approach that is akin to the control group in many of the RCTs we captured for this study. Barriers for routine antenatal care staff include inadequate training, time, resources,^57^ knowledge, skills, and confidence in delivery of lifestyle interventions, which all affect implementation.^57,58^ Antenatal care practitioners will need to be trained to support healthy lifestyle and healthy GWG, integrated with trained staff to deliver evidence-based, cost-effective lifestyle interventions during pregnancy. International, rigorous, evidence-based guidelines are also needed given the inadequacy of the current guidance.^59^ Furthermore, although the focus in pregnancy is on healthy lifestyle and prevention of excessive GWG and not on weight loss, weight stigma remains a major challenge and must be given consideration using appropriate language, resources, and health professional training.^60^

### Strengths and Limitations

This study has some strengths. These included the comprehensive design and inclusion of studies in all languages; with a large sample; and with diverse racial and ethnic populations, settings, countries, and types of interventions. Moreover, the interventions were classified and analyzed by type, advancing the knowledge from previous systematic reviews.

This study also has some limitations. Reporting of lifestyle interventions has inconsistencies and inadequacies, affecting evidence synthesis and strengthening the need for standardization.^61^ Risk of bias was low in 18.8% of studies and unclear in 50.4%. Nine studies on the physical activity intervention, which were captured in the 2017 systematic review, provided limited details on the control group, although all studies included clear physical activity interventions over and above routine care. Framework analysis of behavioral strategies as well as intervention characteristics (ie, intensity, duration, delivery mode, facilitator, and setting), penetration, and participation was beyond the scope of this work, but it is underway. Some outcome definitions and criteria varied, including for gestational diabetes, cesarean section, and admission to NICU, although standardized definitions were applied for preterm birth and SGA and LGA. Aggregate data precluded the analysis of composite outcomes, with multiple outcomes possible in any 1 participant; hence, total adverse maternal and neonatal outcomes were assessed.

## Conclusions

This systematic review and meta-analysis found that antenatal structured diet and physical activity–based lifestyle interventions were associated with reduced GWG and with maternal and neonatal benefits. Structured diet interventions appeared to have greater implications for GWG than physical activity alone or mixed interventions. Diet was associated with improved maternal and neonatal outcomes, whereas physical activity was associated with improved adverse maternal outcomes. Coupled with evidence of cost-effectiveness, this analysis of 117 RCTs involving more than 34 000 women strongly supports the integration of structured diet and physical activity interventions alongside routine antenatal care and policy to improve the health of mothers and their offspring around the world.

## Notes

Supplement. **eTable.** Author, Year, Country Sample Size, Population, Intervention, Comparator and Outcomes of Eligible Studies (By Year of Publication) **eFigure 1.** Funnel Plots and Egger’s Tests Exploring Potential Publication Bias; Gestational Weight Gain (panel A), Maternal and Neonatal Outcomes Across Gestational Diabetes, Hypertensive Disorders of Pregnancy, Preterm Delivery, Cesarean Section, Fetal Death, Small for Gestational Age, Large for Gestational Age and Neonatal Intensive Care admission (panels B-I) **eFigure 2.** Forest Plot of Randomized Controlled Trials and Impact on Gestational Weight Gain
eReferences
