# Supplementary material for: Association of Antenatal Diet and Physical Activity–Based Interventions With Gestational Weight Gain and Pregnancy Outcomes: A Systematic Review and Meta-analysis
Source: JAMA Intern Med. 2021 Dec 20;182(2):1–10. doi: 10.1001/jamainternmed.2021.6373 (PMC8689430; doi:10.1001/jamainternmed.2021.6373)
Supplement: Supplement. — eTable. Author, Year, Country Sample Size, Population, Intervention, Comparator and Outcomes of Eligible Studies (By Year of Publication) eFigure 1. Funnel Plots and Egger’s Tests Exploring Potential Publication Bias; Gestational Weight Gain (panel A), Maternal and Neonatal Outcomes Across Gestational Diabetes, Hypertensive Disorders of Pregnancy, Preterm Delivery, Cesarean Section, Fetal Death, Small for Gestational Age, Large for Gestational Age and Neonatal Intensive Care admission (panels B-I) eFigure 2. Forest Plot of Randomized Controlled Trials and Impact on Gestational Weight Gain eReferences [file jamainternmed-e216373-s001.pdf]

Supplementary Online Content

Teede HJ, Bailey C, Moran LJ, et al. Association of antenatal diet and physical activity–based interventions with gestational weight gain and pregnancy outcomes: a systematic review and meta-analysis. *JAMA Intern Med.* Published online December 20, 2021. doi:10.1001/jamainternmed.2021.6373

eTable. Author, Year, Country Sample Size, Population, Intervention, Comparator and Outcomes of Eligible Studies (By Year of Publication)

eFigure 1. Funnel Plots and Egger's Tests Exploring Potential Publication Bias; Gestational Weight Gain (panel A), Maternal and Neonatal Outcomes Across Gestational Diabetes, Hypertensive Disorders of Pregnancy, Preterm Delivery, Cesarean Section, Fetal Death, Small for Gestational Age, Large for Gestational Age and Neonatal Intensive Care admission (panels B-I)

eFigure 2. Forest Plot of Randomized Controlled Trials and Impact on Gestational Weight Gain

eReferences

This supplementary material has been provided by the authors to give readers additional information about their work.

© 2021 Teede HJ et al. *JAMA Internal Medicine.*

eTable. Author, Year, Country Sample Size, Population, Intervention, Comparator and Outcomes of Eligible Studies (By Year of Publication)

Study	Publication Year	Country	Sample size	Body mass index	Intervention method	Comparison intervention	Outcomes
Gomez Tabarez [1]	1994	Colombia	60	NR	Diet	Standard care	• Macrosomia<br>• Caesarean section<br>• Apgar scores<br>• Infant Birthweight
Lee [2]	1996	UK	370	NR	Physical activity	No details	• Mean duration of labour<br>• Mean pulse rate in labour<br>• Perceived pain level during labour<br>• Birthweight<br>• Incidence of stress continence
Kihlstrand [3]	1999	Sweden	244	NR	Physical activity	No details	• Maternal weight gain<br>• Gestational week at delivery<br>• Weight and height of the neonate<br>• Induced delivery<br>• Mode of delivery<br>• Analgesic methods used during labour<br>• Number of days in the Neonatal Care Unit
Bechtel-Blackwell [4]	2002	USA	46	NR	Diet	Standard care	**Primary**<br>• Cardiorespiratory fitness<br>**Secondary**<br>• Weight gain<br>• Muscular strength and endurance<br>• Physical activity levels (accelerometery and questionnaire)<br>• Maternal glycaemia<br>• Nutrition<br>• Doppler studies of pulsatility index and fetal growth<br>• Sleep quality<br>• Quality of life<br>• Pregnancy outcomes (Birthweight and anthropometric measures, delivery type, pregnancy complications)
**Study**	**Publication Year**	**Country**	**Sample size**	**Body mass index**	**Intervention method**	**Comparison intervention**	**Outcomes**

© 2021 Teede HJ et al. *JAMA Internal Medicine.*

Briley [5]	2002	USA	20	24.0	Mixed	Control group seen twice, visits mirrored first and fifth visits for the intervention but controls had not teaching/ counselling	• Gestational weight gain<br>• Low birthweight
Clapp [6]	2000	USA	46	NR	Physical activity	No detail	• Birthweight<br>• Mid trimester placental growth rate<br>• Placental volume at term<br>• Gestational weight gain
Marquez-Sterling [7]	2000	USA	15	23.7	Physical activity	Individual exercise prescription postpartum	• Gestational weight gain<br>• Skin-fold thickness<br>• Infant birthweight<br>• Apgar scores
Polley [8]	2002	USA	110	27.7	Mixed	Standard care	• Infant birthweight<br>• Low birthweight (<2500g)<br>• Macrosomia (>4000 g)<br>• Weeks of gestation at delivery<br>• Preterm delivery<br>• Caesarean delivery<br>• Pre-eclampsia<br>• Maternal hypertension<br>• GDM
**Study**	**Publication Year**	**Country**	**Sample size**	**Body mass index**	**Intervention method**	**Comparison intervention**	**Outcomes**
Prevedel [9]	2003	Brazil	39	24.7	Physical activity	No intervention	• Weight at baseline (16–20 weeks) and delivery (36–40 weeks)<br>• Preterm delivery

© 2021 Teede HJ et al. *JAMA Internal Medicine.*

							• Birthweight (g)<br>• Large for gestational age<br>• Lean mass<br>• Total fat, relative fat (%)
Garshasbi [10]	2005	Iran	266	25.8	Physical activity	No details	• Low back pain<br>• Lordosis of spine<br>• Weight gain during pregnancy<br>• Pregnancy length (weeks)<br>• Weight of the neonate<br>• Spine flexibility
Khoury [11]	2005	Norway	289	24.3	Diet	Controls advised to have usual diet.<br>Target gain was 8–14kg, intake of fat, carbohydrate and proteins same as intervention	• Gestational age at delivery<br>• Preterm delivery<br>• Maternal weight gain between inclusion and week 30<br>• Preterm stillbirth<br>• Intrauterine growth restriction<br>• Hypertensive complications (pregnancy-induced hypertension/PE)<br>• Fetal distress<br>• Birthweight<br>• Maternal and neonatal lipid profile
Santos [12]	2005	Brazil	90	24.2	Physical activity	No Details	• Low back pain<br>• Maternal weight gain
Sedaghati [13]	2007	Iran	90	24.2	Physical activity	No Details	• Low back pain<br>• Maternal weight gain
**Study**	**Publication Year**	**Country**	**Sample size**	**Body mass index**	**Intervention method**	**Comparison intervention**	**Outcomes**
Baciuk [14]	2008	Brazil	70	NR	Physical activity	No intervention	• Request for analgesia<br>• Caesarean section<br>• Apgar score at 1 minute of ≥ 7<br>• Vaginal delivery<br>• Preterm delivery (< 37 weeks)<br>• Low birthweight (< 2500 g), adequacy of neonatal weight<br>• Gestational age, length of labour (minutes)<br>• Birthweight

© 2021 Teede HJ et al. *JAMA Internal Medicine.*

							• Gestational age<br>• Weight gain<br>• Body fat (%)<br>• Fat-free mass (%)<br>• Body mass index
Barakat [15]	2008	Spain	140	23.8	Physical activity	Women asked to maintain their level of activity	• Gestational weight gain (delivery - pre-pregnancy)<br>• Preterm deliveries<br>• Birthweight<br>• Macrosomia<br>• Birth length<br>• Head circumference<br>• Ponderal index,<br>• Apgar score 1 min,<br>• Apgar score 5 min
Wolff [16]	2008	Denmark	59	34.9	Diet	No intervention	• GDM<br>• Gestational age at delivery<br>• Pregnancy-induced hypertension<br>• Pre-eclampsia<br>• Prolonged pregnancy<br>• Caesarean delivery,<br>• Total gestational weight gain (weight at delivery minus self-reported pre-pregnancy weight)<br>• Weight gain from 15 weeks to 36 weeks<br>• Birthweight<br>• Placental weight<br>• Infant length<br>• Head circumference<br>• Abdominal circumference
**Study**	**Publication Year**	**Country**	**Sample size**	**Body mass index**	**Intervention method**	**Comparison intervention**	**Outcomes**
Asbee [17]	2009	USA	100	26.1	Diet with physical activity	Standard care	**Primary**<br>• Rate of adherence to the IOM guidelines between our study groups<br>**Secondary**<br>• Mode of delivery<br>• Rate of operative vaginal delivery<br>• Neonatal weight<br>• Incidence of pre-eclampsia<br>• GDM<br>• Vaginal/perineal lacerations

© 2021 Teede HJ et al. *JAMA Internal Medicine.*

							• Shoulder dystocia
Jeffries [18]	2009	Australia	282	25.7	Mixed	No intervention	• Gestational weight gain: weekly and total\, 11 weeks to delivery and compliance with IOM recommendations<br>• Birthweight<br>• Small for gestational age and large for gestational age (weight < 10 centile and > 90 centile)<br>• Preterm delivery<br>• Instrumental delivery<br>• Caesarean delivery<br>• Pre-eclampsia<br>• Pregnancy-induced hypertension<br>• GDM<br>• Apgar score at 5 minutes of < 7<br>• Hypoglycaemia<br>• Shoulder dystocia<br>• Gestational age at delivery
Ong [19]	2009	Australia	12	36.0	Physical activity	No intervention	• Weight gain from 18 to 28 weeks' gestation<br>• Post-intervention glucose and insulin levels on oral glucose tolerance test
Thornton [20]	2009	USA	232	37.8	Diet	Standard care	**Primary**<br>• Compare perinatal outcomes control vs the study groups<br>**Secondary**<br>• Compare adherent and non-adherent women in the study group
**Study**	**Publication Year**	**Country**	**Sample size**	**Body mass index**	**Intervention method**	**Comparison intervention**	**Outcomes**
Guelinckx [21]	2010	Belgium	195	33.6	Mixed	No intervention	• Pregnancy-induced hypertension, pre-eclampsia, chronic hypertension<br>• Gestational weight gain in accordance with IOM<br>• Gestational weight gain > 11.2 kg (weight gain from pre-pregnancy to 38 weeks)<br>• Gestational age at delivery<br>• Induction of labour<br>• Caesarean section<br>• Birthweight/length<br>• Macrosomia (birthweight > 4000 g)<br>• Total physical activity score

© 2021 Teede HJ et al. *JAMA Internal Medicine.*

Hopkins [22]	2010	New Zealand	84	25.5	Physical activity	Controls asked to continue usual daily activities during pregnancy	• Maternal insulin sensitivity<br>• Neonatal auxology<br>• Body composition<br>• Growth-related peptides in cord blood
Khaledan [23]	2010	Iran	39	28.3	Physical activity	No intervention	• Gestational age at delivery<br>• Caesarean section<br>• Neonatal weight<br>• Weight 1 and 2 months post intervention 28 to 36 weeks
Barakat [24]	2011	Spain	67	NR	Physical activity	Standard care	• Maternal perception of health status (Short Form questionnaire-36 items King's Health questionnaire)<br>• Frequency of urine incontinence (CIQ-SF incontinence classification<br>• Gestational weight gain<br>• Gestational age at delivery<br>• Mode of delivery (normal, instrumental, Caesarean)<br>• Delivery lacerations type<br>• Systolic and diastolic blood pressure<br>• 1-hour glucose level<br>• Birthweight<br>• Macrosomia<br>• Apgar score at 1 minute<br>• Apgar score at 5 minutes
**Study**	**Publication Year**	**Country**	**Sample size**	**Body mass index**	**Intervention method**	**Comparison intervention**	**Outcomes**
Haakstad [25]	2011	Norway	101	25.3	Physical activity	Participants were neither encouraged nor discouraged from exercising	• Gestational weight gain (weight after completion of intervention at around 37 weeks minus self-reported pre-pregnancy weight)<br>• Weight gain as per IOM categories<br>• Postpartum weight retention<br>• Skin fold thickness
Huang [26]	2011	Taiwan	189	21.0	Mixed	Face-to-face nurse education on concerns, written pregnancy	• Body weight<br>• Lifestyle behaviours<br>• Self-efficacy<br>• Body image<br>• Depression and social support

© 2021 Teede HJ et al. *JAMA Internal Medicine.*

						general nutrition and exercise information	
Jackson [27]	2011	USA	287	27	Mixed	Standard care	**Primary**<br>• Self-reported servings per day or week of healthful foods (e.g. fruits and vegetables) and unhealthful foods<br>• Exercise duration and frequency.<br>**Secondary**<br>• Food knowledge<br>• Knowledge of weight gain guidelines<br>• Weight gain above the IOM guidelines
Nascimento [28]	2011	Brazil	82	36.9	Physical activity	Standard antenatal advice and standard nutritional counselling No specific physical activity counselling	**Primary**<br>• Gestational weight gain<br>• Excessive maternal weight gain<br>**Secondary**<br>• Increased blood pressure<br>• Caesarean section,<br>• Birthweight, gestational age at delivery<br>• Preterm delivery<br>• Apgar scores (1 and 5 minutes),<br>• Large and small for gestational age<br>• Quality of life (WHO Quality of Life survey)
**Study**	**Publication Year**	**Country**	**Sample size**	**Body mass index**	**Intervention method**	**Comparison intervention**	**Outcomes**
Phelan [29]	2011	USA	393	27.4	Mixed	Standard visits and nutrition counselling, brief face-to-face with study intervention team at recruitment, general newsletters, regular weighing, no graphs	**Primary:**<br>• Proportion with excess gestational weight gain on IOM<br>• Proportion ± 9 kg or below pre-pregnancy weight at 6 months postpartum<br>**Secondary:**<br>• GDM<br>• Maternal hypertension<br>• Pre-eclampsia<br>• Gestational age at delivery<br>• Preterm delivery<br>• Caesarean section<br>• Infant birthweight<br>• Low birthweight<br>• Macrosomia

© 2021 Teede HJ et al. *JAMA Internal Medicine.*

Quinlivan [30]	2011	Australia	124	NR	Diet	Standard care	**Primary**<br>• GDM<br>**Secondary**<br>• Gestational weight gain<br>• Neonatal Birthweight
Vinter [31]	2011	Denmark	304	34.3	Diet with physical activity	Information on study purpose and content Website diet and physical activity in pregnancy	**Primary**<br>• Gestational weight gain (35 weeks- weight at inclusion)<br>• Pre-eclampsia<br>• Pregnancy-induced hypertension<br>• GDM<br>• Caesarean section<br>• Macrosomia/large for gestational age<br>• Admission to the NICU
Barakat, Pelaez [32]	2012	Spain	290	22.9	Physical activity	No details	• Maternal age<br>• Body mass index<br>• Smoking and alcohol intake<br>• Occupational activity, standing and domestic tasks time<br>• Gestational age<br>• Mode of delivery<br>• Blood pressure<br>• Birthweight<br>• Apgar score
**Study**	**Publication Year**	**Country**	**Sample size**	**Body mass index**	**Intervention method**	**Comparison intervention**	**Outcomes**
Barakat, Cordero [33]	2012a	Spain	83	24.4	Physical activity	Standard care	**Primary**<br>• GDM<br>**Secondary**<br>• Birthweight<br>• Risk of macrosomia<br>• Gestational age<br>• Risk of caesarean delivery<br>• Maternal weight gain
de Oliveria Melo [34]	2012	Brazil	171	23.9	Physical activity	Standard care	• Pre-eclampsia<br>• Fetal macrosomia<br>• Birthweight<br>• Large for gestational age<br>• Small for gestational age<br>• Maximal oxygen consumption (VO2max)<br>• pulsatility index of the uterine, umbilical, and middle cerebral arteries

© 2021 Teede HJ et al. *JAMA Internal Medicine.*

Hui [35]	2012	Canada	183	NR	Diet with physical activity	Standard care from National guidelines No exercise or diet intervention	• Excessive weight gain<br>• Intake<br>• Physical activity<br>• Large for gestational age<br>• GDM<br>• Weight-related obstetric procedures<br>• Gestational weight gain<br>• Birthweight
Korpi-Hyövälti [36]	2012	Finland	54	26.4	Diet	General nurse session; verbal and written on diet and physical activity on GDM Prevention	**Primary**<br>• GDM<br>**Secondary**<br>• Nutrient intake<br>• Weight gain<br>• Birthweight
**Study**	**Publication Year**	**Country**	**Sample size**	**Body mass index**	**Intervention method**	**Comparison intervention**	**Outcomes**
Oostdam [37]	2012	Netherlands	105	35.6	Physical activity	Standard care by midwives and obstetricians	**Primary**<br>• Fasting plasma glucose and relative increase in insulin resistance in mother<br>• Neonatal birthweight<br>**Secondary**<br>• Maternal serum triglycerides, high-density lipoprotein, cholesterol and HbA1c<br>• Gestational weight gain<br>• Maternal physical activity level<br>• Fetal growth<br>• Changes in health-care and non-health-care costs
Price [38]	2012	USA	62	27.7	Physical activity	Told not to exercise and confirmed with completers every 6 weeks	**Primary**<br>• Length of pregnancy<br>• New born Birthweight<br>• Postpartum recovery<br>**Secondary**<br>• Strength, flexibility, musculoskeletal discomforts<br>• Incidence of GDM and gestational hypertension

© 2021 Teede HJ et al. *JAMA Internal Medicine.*

							• Length of first and second stages of labor<br>• Frequency of caesarean section<br>• Frequency of assisted delivery<br>• New born Apgar scores<br>• Placenta weight<br>• Postpartum weight retention
Rakhshani [39]	2012	India	68	25.2	Physical activity	Standard care plus conventional antenatal exercises (walking)	**Primary**<br>• Study feasibility<br>• Hypertensive disorders of pregnancy<br>• Intrauterine growth restriction<br>• Preterm deliveries<br>**Secondary**<br>• Apgar-1 and Apgar-5 scores<br>• Small for gestational age<br>• Large for gestational age<br>• Large birth weight
Ramírez-Vélez [40]	2012	Colombia	50	21.9	Physical activity	Standard care	• Endothelial function on flow-mediated dilatation<br>• Cardiorespiratory fitness on VO2max in 6-min walk test
**Study**	**Publication Year**	**Country**	**Sample size**	**Body mass index**	**Intervention method**	**Comparison intervention**	**Outcomes**
Stafne [41]	2012	Norway	854	24.9	Physical activity	Standard care, Written Information on diet, pelvic floor exercises pelvic pain	**Primary**<br>• Prevalence of GDM at 32–36 weeks' gestation<br>• Insulin resistance; homeostasis model<br>**Secondary**<br>• Maternal weight at follow-up<br>• Weight gain at follow-up<br>• Body mass index at follow-up<br>• Pre-eclampsia<br>• Gestational hypertension<br>• Caesarean delivery<br>• Operative vaginal delivery<br>• Gestational age at delivery<br>• Birthweight ≥ 4000 g<br>• Apgar score<br>• Admission to the NICU
Walsh [42]	2012	Ireland	759	27.1	Diet	Routine antenatal care with no specific dietary	**Primary**<br>• Mean birthweight centiles and ponderal indices (14, 28, 34 weeks, birth and 3 months postpartum)<br>**Secondary**

© 2021 Teede HJ et al. *JAMA Internal Medicine.*

Study	Publication Year	Country	Sample size	Body mass index	Intervention method	Comparison intervention	Outcomes
						recommendation or advice about gestational weight gain	• Maternal weight gain (14, 28 and 34 weeks gestation, birth and 3 months postpartum)<br>• Adherence to IOM recommendations for gestational weight gain<br>• Maternal glucose intolerance
Althuizen [43]	2013	Netherlands	269	27.6	Mixed	Standard care	**Primary**<br>• Change in body weight and BMI (15, 25 and 35 weeks of pregnancy and postpartum)<br>• Skin fold thickness and body fat percentage<br>**Secondary**<br>• Physical activity by Short Questionnaire to Assess Health enhancing physical activity (SQUASH) and accelerometer data<br>• Questionnaire for nutrition and related behaviours (Dutch eating behaviour questionnaire)<br>• Leptin, ghrelin, fasting glucose, insulin, cortisol, IGF-1, IGF binding proteins in a subgroup, cord blood.
**Study**	**Publication Year**	**Country**	**Sample size**	**Body mass index**	**Intervention method**	**Comparison intervention**	**Outcomes**
Barakat [44]	2013	Spain	279	23.9	Physical activity	Standard care	• Mode of delivery (normal, instrumental, Caesarean)<br>• Gestational age at delivery<br>• Preterm delivery (< 37 weeks)<br>• Maternal weight gain<br>• Blood pressure<br>• 1-hour glucose tolerance test<br>• GDM<br>• Birthweight/length<br>• Ph of the umbilical cord blood<br>• Apgar score
Bogaerts [45]	2013	Belgium	197	34.7	Mixed	Routine antenatal care as per national guideline	• Gestational weight gain compared to self-reported pre-pregnancy weight; total at delivery, first trimester at 14 weeks, second trimester at 22 weeks, third trimester at 34 weeks<br>• Anxiety (State and Trait Anxiety Inventory)<br>• Depression (Edinburgh Postnatal Depression Scale)<br>• Pregnancy-induced hypertension<br>• Pre-eclampsia<br>• GDM<br>• Induction of labour<br>• Method of delivery (vaginal, vacuum/forceps, elective/ emergency Caesarean section)

© 2021 Teede HJ et al. *JAMA Internal Medicine.*

							• Birthweight<br>• Apgar score at 1 and 5 minutes
Deveer [46]	2013	Turkey	100	28.6	Diet	Standard care	• Birthweight<br>• Gestational age at delivery<br>• Total maternal weight gain<br>• Large for gestational age<br>• Macrosomia (> 4000 g)<br>• Small for gestational age<br>• Caesarean delivery<br>• Preterm delivery<br>• NICU admission<br>• Antenatal pre-eclampsia<br>• Perineal trauma<br>• Postpartum atonia
**Study**	**Publication Year**	**Country**	**Sample size**	**Body mass index**	**Intervention method**	**Comparison intervention**	**Outcomes**
Harrison	2013	Australia	238	31.4	Mixed	Single brief session verbal and written Diet and Physical Activity Guideline information. Weight gain not mentioned	**Primary**<br>• Gestational weight gain (baseline; 12, 16 and 28 weeks)<br>**Secondary**<br>• GDM (Australian Diabetes in Pregnancy Soc, International Association of the Diabetes and Pregnancy Study Groups)<br>• Physical activity using pedometer and International Physical Activity Questionnaire<br>• Risk perception for GDM development and excess gestational weight gain (four-point Likert scale adapted from the theory of health stage of change was used)
Ruiz [47]	2013	Spain	927	NR	Physical activity	Standard care, information provided on nutrition and physical activity counselling and not discouraged from exercising	**Primary**<br>• Gestational weight gain (clinic predelivery -first visit)<br>**Secondary**<br>• GDM<br>• Hypertension<br>• Gestational age at delivery<br>• Mode of delivery (natural, instrumental or Caesarean)<br>• Time of dilatation, Expulsion and childbirth<br>• Birthweight<br>• Low birthweight<br>• Macrosomia
Tomic [48]	2013	Croatia	334	23.0	Physical activity	Standard care	**Primary**<br>• Intrauterine growth restriction

© 2021 Teede HJ et al. *JAMA Internal Medicine.*

Study	Publication Year	Country	Sample size	Body mass index	Intervention method	Comparison intervention	Outcomes
							• Excessive fetal growth (macrosomia)<br>**Secondary**<br>• Pre-eclampsia, Pregnancy-induced hypertension<br>• GDM,<br>• Mode of delivery
Barakat [49]	2014	Spain	200	23.9	Physical activity	Standard care	• Gestational age<br>• Maternal weight gain<br>• Maternal weight gain (IOM guidelines)<br>• Body mass index<br>• Smoking habits<br>• Mode of delivery<br>• Blood pressure during pregnancy
Di Carlo [50]	2014	Italy	120	25.8	Diet	Standard written diet advice in pregnancy	**Primary**<br>• Gestational weight gain (between baseline and term)<br>**Secondary**<br>• Gestational weight gain (pre-pregnancy and term)<br>• Birthweight
Dodd [51]	2014	Australia	2199	32.5	Mixed	Standard hospital guidelines, with no routine provision of dietary, lifestyle and behavioural recommenda tions	**Primary**<br>• Large for gestational age infant; ≥ 90th centile<br>**Secondary**<br>• Preterm delivery (< 37 weeks' gestation)<br>• Mortality (stillbirth or infant death)<br>• Death of a live-born infant before discharge, with no lethal congenital anomalies<br>• Congenital anomalies<br>• Infant birthweight ≥ 4000g<br>• Hypoglycaemia with intravenous treatment<br>• Admission to the NICU or special care baby unit<br>• Hyperbilirubinemia requiring phototherapy<br>• Nerve palsy or Fracture<br>• Birth trauma<br>• Shoulder dystocia<br>• Maternal hypertension and pre-eclampsia<br>• Maternal GDM<br>• Antenatal hospital stay<br>• Antepartum haemorrhage requiring hospitalisation<br>• Preterm prelabour ruptured membranes<br>• Chorioamnionitis requiring antibiotic use during labour<br>• Need and reason for induction of labour<br>• Any antibiotic use during labour

© 2021 Teede HJ et al. *JAMA Internal Medicine.*

							• Caesarean section<br>• Postpartum haemorrhage (blood loss ≥ 600 ml)<br>• Perineal trauma, wound infection<br>• Endometritis<br>• Use of postnatal antibiotics<br>• Length of postnatal hospital stay<br>• Thromboembolic disease<br>• Maternal death
**Study**	**Publication Year**	**Country**	**Sample size**	**Body mass index**	**Intervention method**	**Comparison intervention**	**Outcomes**
Hui [52]	2014	Canada	113	NR	Diet with physical activity	Standard care as per national guidelines, information on physical activity and healthy eating in pregnancy from Health Canada	• Delivery route<br>• Maternal weight gain<br>• Excessive Gestational weight gain<br>• Birthweight<br>• Birthweight-related obstetric procedures (induction, forceps or caesarean section)<br>• GDM<br>• Body mass index<br>• Large for gestational age<br>• Physical activity levels<br>• Food intakes
Ko [53]	2014	USA	1196	25.7	Physical activity	Standard care	• Physical activity levels<br>• Presence of gallbladder sludge or stones at 18 or 36 weeks<br>• Glucose and lipid levels<br>• GDM<br>• Birthweight<br>• Gestational age<br>• Gestational weight gain
Kong [54]	2014	USA	37	30.7	Physical activity	No details	• Physical activity measures<br>• Gestational weight gain<br>• Gestational weight gain exceeding IOM guidelines<br>• Birthweight<br>• Gestational length at delivery<br>• Birthweight z score<br>• Low Birthweight ≤2500 g<br>• Macrosomia<br>• Apgar score 1 min and 5 min<br>• Preterm delivery

© 2021 Teede HJ et al. *JAMA Internal Medicine.*

							• Caesarean delivery<br>• Pre-eclampsia<br>• Maternal hypertension<br>• GDM
**Study**	**Publication Year**	**Country**	**Sample size**	**Body mass index**	**Intervention method**	**Comparison intervention**	**Outcomes**
Li [55]	2014	China	239	NR	Physical activity	No details	• Duration of labour<br>• Caesarean rate<br>• New born weight<br>• Mean body weight gain<br>• Labour pain perception<br>• Neonatal weight<br>• Neonatal birth condition
Petrella[56]	2014	Italy	61	33.8	Diet with physical activity	Simple nutritional booklet on national guidelines for healthy diet in pregnancy	**Primary:**<br>• Rate of women with weight gain exceeding the ranges recommended by IOM for each body mass index category<br>**Secondary:**<br>• Diagnoses of GDM<br>• Gestational hypertension<br>• Rate of preterm delivery
Renault [57]	2014 Mix	Denmark	425	34.6	Mixed	Standard care with a consult with a dietitian at 11–14 weeks Dietary advice as per national guidelines for healthy eating. Verbal advice only, aiming for a gestational weight gain of < 5 kg	**Primary**<br>• Gestational weight gain (weight at 36–37 weeks minus self-reported pre-pregnancy weight)<br>**Secondary**<br>• GDM (oral glucose tolerance test at 17–20 weeks and 27–30 weeks)<br>• Gestational hypertension<br>• Pre-eclampsia<br>• Induction of labour<br>• Caesarean section (emergency/planned)<br>• Gestational age at delivery<br>• Preterm delivery (28–34 weeks and 34–37 weeks)<br>• Fetal birthweight<br>• Relative birthweight<br>• Small for gestational age<br>• Large for gestational age<br>• Macrosomia

© 2021 Teede HJ et al. *JAMA Internal Medicine.*

							• Ph of umbilical cord blood<br>• Placental weight
**Study**	**Publication Year**	**Country**	**Sample size**	**Body mass index**	**Intervention method**	**Comparison intervention**	**Outcomes**
Vesco [58]	2014	USA	114	36.7	Diet with physical activity	Onetime dietary advice	**Primary**<br>• Gestational weight gain<br>**Secondary**<br>• Gestational hypertension/pre-eclampsia<br>• GDM<br>• Mode of delivery (caesarean section versus vaginal)<br>• Preterm delivery<br>• Neonatal hypoglycaemia<br>• Hyperbilirubinemia<br>• Respiratory morbidities<br>• Admissions to the special care nursery or neonatal intensive care unit<br>• Perinatal mortality
Bisson [59]	2015	Canada	45	34.75	Physical activity	Usual activities, no limits on physical activity. Pamphlet (from Kino-Québec, an agency) on physical activity and exercises for pregnancy	**Primary**<br>• Physical activity levels- accelerometery at 14, 28 and 36 weeks of gestation<br>**Secondary**<br>• Weight gain from 14 to 36 weeks<br>• Weight gain from 14 to 28 weeks<br>• Total gestational weight gain<br>• Dietary intakes at 14 and 28 weeks of gestation<br>• Neonatal anthropometry<br>• Birthweight
Cordero [60]	2015	Spain	257	23.05	Physical activity	Standard care	**Primary**<br>• GDM<br>**Secondary**<br>• Excess weight gain on pre-pregnancy body mass index<br>• Gestational age at delivery<br>• Mode of delivery<br>• Macrosomia (>4000 g)<br>• Low-birthweight (<2500 g)<br>• Length of new born

© 2021 Teede HJ et al. *JAMA Internal Medicine.*

Study	Publication Year	Country	Sample size	Body mass index	Intervention method	Comparison intervention	Outcomes
Dekker [61]	2015	Australia	35	36.8	Physical activity	Standard care	• Gestational weight gain<br>• Gestational weight gain exceeding IOM guidelines<br>• BMI<br>• Systolic and diastolic BP<br>• blood glucose, insulin, triglyceride, total, HDL & LDL cholesterol level<br>• C-section<br>• Gestational age delivery<br>• Birth weight and length<br>• Cord glucose<br>• Cord insulin<br>• Cord Cholesterol<br>• Cord Triglycerides<br>• Cord HDL cholesterol<br>• Cord LDL cholesterol
Gesell [62]	2015	USA	87	NR	Diet with physical activity	Standard care	**Primary**<br>• Gestational weight exceeding IOM recommendations<br>**Secondary**<br>• Birthweight<br>• Gestational age at birth
Hawkins [63]	2015	USA	68	NR	Mixed	Standard care	**Primary**<br>• Physical activity<br>• Diet (caloric intake and percentage of calories from fat)<br>**Secondary**<br>• Gestational weight gain<br>• Infant birthweight<br>• Biomarkers associated with insulin resistance
Jing [64]	2015	China	221	20.59	Mixed	Standard care	• Total gestational weight gain<br>• Gestational weight gain exceeding IOM guidelines<br>• Dietary intake<br>• Physical activity levels

© 2021 Teede HJ et al. *JAMA Internal Medicine.*

Study	Publication Year	Country	Sample size	Body mass index	Intervention method	Comparison intervention	Outcomes
Perales [65]	2015	Spain	167	NR	Physical activity	Standard care	• Center for Epidemiologic Studies Depression Scale at 9–12 weeks and end of pregnancy<br>• Gestational weight gain<br>• Percentage with excess weight gain (IOM guidelines)<br>• Percentage with adequate weight gain (IOM guidelines)<br>• Gestation age at delivery<br>• Mode of delivery (normal, instrumental, Caesarean)<br>• Birthweight<br>• Length of the baby at birth<br>• Head circumference<br>• Apgar score at 1 minute<br>• Apgar score at 5 minutes
Petrov Fieril [66]	2015	Sweden	72	22.8	Physical activity	Generalized exercise recommendation, home-based training program and phone follow up	• Health-related quality of life<br>• Physical strength<br>• Pain,<br>• Gestational weight gain<br>• Blood pressure<br>• Functional status<br>• Activity level<br>• Perinatal data
Poston [67]	2015	UK	1554	36.3	Mixed	Routine antenatal care, explaining the risks of obesity, advising on healthy diet and safe levels of physical activity	**<u>Primary</u>**<br>• Diagnosis of GDM according to International Association of the Diabetes and Pregnancy Study Groups criteria<br>• Large for gestational age baby (> 90th weight centile)<br>**<u>Secondary</u>**<br>• Pre-eclampsia<br>• Mode of delivery<br>• Induction of labour<br>• Blood loss at delivery<br>• Inpatient nights<br>• Gestational weight gain<br>• Fasting glucose, insulin, insulin resistance at 28 weeks' gestation<br>• Referral to antenatal clinic after oral glucose tolerance test<br>• Fetal growth at 28 weeks' gestation
**Study**	**Publication Year**	**Country**	**Sample size**	**Body mass index**	**Intervention method**	**Comparison intervention**	**Outcomes**

© 2021 Teede HJ et al. *JAMA Internal Medicine.*

							• Insulin or metformin treatment in pregnancy<br>• Quality of life<br>• Anthropometry; mid-arm, hip, thigh, skin fold thickness<br>• Fructosamine, lipid profile<br>• Epigenetic, urinary and metabolomic biomarkers<br>• Diet and physical activity<br>• Depression<br>• Smoking<br>• Birthweight of baby<br>• Gestational age at delivery<br>• Neonatal death<br>• Neonatal complications<br>• Baby's anthropometry; head, abdomen, skin folds<br>• Epigenetic and other markers<br>• Infant feeding habits and anthropometry at 6 months
Ronnberg [68]	2015	Sweden	374	25.3	Physical activity	Standard care	• Proportion of women gaining above IOM recommendations on gestational weight gain
Aşcı [69]	2016	Turkey	90	23.3	Mixed	Standard care	**Primary**<br>• Gestational weight gain<br>• Proportion of pregnant women with gestational weight gain within the Institute of Medicine (IOM) guidelines<br>**Secondary**<br>• Lifestyle behaviours<br>• Dietary habits<br>• Postpartum weight retention
Barakat [70]	2016	Spain	765	23.5	Physical activity	Standard care	**Primary**<br>• incidence of hypertension during pregnancy<br>**Secondary**<br>• Excessive gestational weight<br>• Incidence of developing GDM<br>• Delivering a preterm infant<br>• length of new born<br>• Apgar scores at 1 and 5 minutes after delivery<br>• Cord blood pH<br>• Macrosomia (>4000 g)<br>• Low-birthweight (<2500 g) infant
**Study**	**Publication Year**	**Country**	**Sample size**	**Body mass index**	**Intervention method**	**Comparison intervention**	**Outcomes**
Garnaes [71]	2016	Norway	74	34.5	Physical activity	Standard care	**Primary**<br>• Gestational weight gain from baseline testing to delivery

© 2021 Teede HJ et al. *JAMA Internal Medicine.*

							**Secondary**<br>• Body mass index<br>• Body composition<br>• Physical activity level<br>• Skinfold thickness<br>• Blood pressure<br>• Various blood tests<br>• Incidence of GDM<br>• Incidence of maternal hypertension in late pregnancy
Herring [72]	2016	USA	56	32.9	Mixed	Standard care	• Gestational weight gain and exceeding IOM guidelines<br>• Birthweight<br>• Small-for-gestational-age<br>• Large-for-gestational-age<br>• Mode of delivery<br>• GDM
Koivusalo [73]	2016	Finland	269	32.3	Diet with physical activity	Standard care	**Primary**<br>• GDM<br>**Secondary**<br>• Fasting plasma glucose concentrations<br>• Weight change<br>• Incidence of pre-eclampsia and gestational hypertension<br>• Mode of delivery
McCarthy [74]	2016	Australia	371	30.3	Mixed	Standard care	**Primary**<br>• Gestational hypertension and pre-eclampsia<br>• Diabetes<br>• Assisted or caesarean birth<br>• Shoulder dystocia, severe perineal trauma<br>• Postpartum haemorrhage<br>• Maternal high dependency care<br>**Secondary**<br>• Gestational weight gain at 36 weeks' gestation<br>• Quality of life<br>• Maternal serum levels of 28-week leptin, adiponectin and C-reactive protein
**Study**	**Publication Year**	**Country**	**Sample size**	**Body mass index**	**Intervention method**	**Comparison intervention**	**Outcomes**
Perales [75]	2016	Spain	166	NR	Physical activity	Standard care	• Duration of stages of labour<br>• Gestational weight gain<br>• Percentage with excess weight gain (IOM guidelines)<br>• Percentage with adequate weight gain (IOM guidelines)

© 2021 Teede HJ et al. *JAMA Internal Medicine.*

							• Gestation age at delivery<br>• Mode of delivery (normal, instrumental)<br>• Birthweight<br>• Birth length<br>• Head circumference<br>• Apgar score at 1 minute<br>• Apgar score at 5 minutes<br>• Ph of umbilical cord
Perales, Santos-Lozano [76]	2016a	Spain	142	NR	Physical activity	Standard care	• Type (normal, instrumental, caesarean)<br>• Duration of delivery, occurrence of preterm delivery<br>• New born gestational age<br>• New born weight, height and head circumference at birth<br>• Apgar score at 1 and 5 min<br>• Ph of the umbilical cord
Seneviratne [77]	2016	New Zealand	75	33.1	Physical activity	Standard care	**Primary**<br>• Offspring birthweight<br>**Secondary**<br>• Pre-specified maternal and perinatal parameters
Smith [78]	2016	USA	45	26.4	Mixed	Standard care	• Maternal Anthropometric Data<br>• Gestational weight gain<br>• Physical Activity<br>• Dietary Intake
Sun [79]	2016	China	66	26.7	Diet with physical activity	Physical activity, diet weight gain counselling at 8–12 weeks and standard pregnancy education	**Primary**<br>• GDM<br>**Secondary**<br>• Gestational weight gain
**Study**	**Publication Year**	**Country**	**Sample size**	**Body mass index**	**Intervention method**	**Comparison intervention**	**Outcomes**
Toosi [80]	2016	Iran	120	NR	Physical activity	Standard care	• Length of pregnancy<br>• Delivery phases<br>• Mode of delivery<br>• Apgar score<br>• Infant weight, height and head circumference

© 2021 Teede HJ et al. *JAMA Internal Medicine.*

Wang [81]	2016	China	226	26.785	Physical activity	Standard care	**Primary**<br>• GDM<br>**Secondary**<br>• Gestational weight gain<br>• Insulin resistance levels at 36 gestational weeks<br>• Hypertensive disorders of pregnancy<br>• Caesarean delivery<br>• Mean gestational age at birth<br>• Preterm delivery<br>• Macrosomia and large-for-gestational-age infants
Assaf-Balut	2017	Spain	874	23.9	Diet	Standard care	**Primary**<br>• GDM in women with past normal fasting glucose<br>**Secondary**<br>• Percent of diabetic women requiring insulin therapy<br>• Gestational weight gain<br>• Pregnancy-induced hypertension<br>• Caesarean section<br>• Perineal trauma and Shoulder dystocia<br>• Preterm delivery (< 37 GW)<br>• Neonates small for gestational age (10th percentile)<br>• Admissions to the NICU
Bruno [82]	2017	Italy	131	34.2	Diet with physical activity	Simple book on diet and physical activity from national guidelines in pregnancy	**Primary**<br>• GDM<br>**Secondary**<br>• Gestational weight gain<br>• Rate of pregnancy-induced hypertension<br>• Preterm delivery<br>• Mode of delivery<br>• Birthweight and its distribution<br>• Apgar score at 5 min<br>• Need for resuscitation and NICU admission
**Study**	**Publication Year**	**Country**	**Sample size**	**Body mass index**	**Intervention method**	**Comparison intervention**	**Outcomes**
Chao	2017	USA	38	31.2	Diet with physical activity	Standard care	• Gestational weight gain<br>• Glucose<br>• Gestational week at delivery<br>• Birthweight<br>• 5-min Apgar scores
da Silva	2017	Brazil	594	25.2	Physical activity	Standard care	**Primary**<br>• Preterm delivery

© 2021 Teede HJ et al. *JAMA Internal Medicine.*

							• Pre-eclampsia<br>**Secondary**<br>• Gestational weight gain<br>• GDM<br>• Birthweight<br>• Infant length and head circumference
Daly [83]	2017	Ireland	76	34.7	Physical activity	Standard care	**Primary**<br>• Mean fasting glucose (24–28 weeks of gestation)<br>**Secondary**<br>• Longitudinal fasting plasma glucose concentrations<br>• Incidence of GDM on OGTT at 24–28 weeks<br>• Birth outcomes<br>• Induction of labour<br>• Mode of delivery and length of labour<br>• Birthweight, centile l<10th and > 90th centiles<br>• Gestational age at delivery<br>• Preterm delivery<br>• Admission to NICU<br>• Apgar scores <7 at 1 and 5 minutes<br>• Gestational weight gain: 24–28 and 36 weeks<br>• Excessive gestational weight gain >9.1 kg at 36 weeks<br>• Mean postpartum weight retention at 6 weeks
Van Horn [84]	2017	USA	280	31.0	Diet with physical activity	Standard care	**Primary**<br>• Gestational weight gain<br>**Secondary**<br>• Weekly rate of gestational weight gain<br>• New born anthropometrics<br>• Maternal diet quality and physical activity<br>• Blood pressure
**Study**	**Publication Year**	**Country**	**Sample size**	**Body mass index**	**Intervention method**	**Comparison intervention**	**Outcomes**
Sagedal [85]	2017	Norway	600	25.6	Diet with physical activity	Standard care	• Maternal weight gain and postpartum weight retention<br>• Body composition at 36 weeks of gestation<br>• Infant birthweight and percent large for gestational age (> 90th percentile) infants<br>• Maternal glucose and related hormones<br>• Incidence of operative deliveries and complications
Sewell	2017	UK	28	NR	Diet	Standard care	• Urinary biomarkers<br>• Mediterranean diet score

© 2021 Teede HJ et al. *JAMA Internal Medicine.*

							• Gestational weight gain
Simmons [86]	2017 HE	UK	436	36	Mixed	Standard care	• Gestational weight gain at 35 to 37 weeks<br>• Fasting glucose<br>• Insulin sensitivity
Willcox [87]	2017	Australia	91	31	Mixed	Standard care	**<u>Primary</u>**<br>• Intervention feasibility<br>**<u>Secondary</u>**<br>• Gestational weight gain<br>• Self-reported dietary intake<br>• Physical activity
Abdel-Aziz	2018	Egypt	147	NR	Diet with physical activity	Standard care	• Body mass index<br>• Excessive gestational weight gain (IOM guidelines)<br>• Anaemia<br>• GDM<br>• Pregnancy-induced hypertension<br>• Caesarean section<br>• Macrosomia<br>• Preterm
Bacchi	2018	Argentina	111	23.55	Physical activity	Standard care	**<u>Primary</u>**<br>• Total maternal weight gain during pregnancy (kg)<br>• Birthweight (g) measured at the first prenatal visit<br>**<u>Secondary</u>**<br>• Gestational age (days)<br>• Maternal blood pressure<br>• Infant length<br>• Head circumference<br>• Apgar scores
**Study**	**Publication Year**	**Country**	**Sample size**	**Body mass index**	**Intervention method**	**Comparison intervention**	**Outcomes**
Barakat	2018	Spain	325	NR	Physical activity	Standard care	**<u>Primary</u>**<br>• Length of the stages of labour<br>**<u>Secondary</u>**<br>• Mode of delivery<br>• Gestational age<br>• Maternal weight gain<br>• Preterm delivery<br>• Use of epidural<br>• Birthweight<br>• Apgar scores

© 2021 Teede HJ et al. *JAMA Internal Medicine.*

							• Arterial cord pH
Cahill	2018	USA	240	32.4	Mixed	Standard physical activity program	**Primary**<br>• Gestational weight gain exceeding IOM guidelines<br>**Secondary**<br>• Weekly and total Gestational weight gain<br>• Body fat and fat-free masses<br>• Indices of glycaemic control<br>• Plasma lipid profile<br>• Systolic and diastolic blood pressures<br>• GDM<br>• Hypertensive disease of pregnancy<br>• Preterm delivery<br>• Fetal death<br>• Neonatal Birthweight and length<br>• Neonatal body composition (fat-free mass and percent body fat)<br>• Large for gestational age<br>• Small for gestational age<br>• Umbilical cord plasma glucose and insulin concentrations<br>• Medical complications (neonatal intensive care unit admission within 24 hours of life, respiratory distress syndrome, hypoglycaemia [plasma glucose< 30 mg/dL at any time], and neonatal death within the first 28 days of life)
**Study**	**Publication Year**	**Country**	**Sample size**	**Body mass index**	**Intervention method**	**Comparison intervention**	**Outcomes**
Chan	2018	China	229	23.62	Diet with physical activity	Standard care	**Primary**<br>• Proportion of women developing GDM<br>**Secondary**<br>• Large for gestational age<br>• Macrosomia (>4000 g)<br>• Gestational weight gain<br>• Pregnancy-induced hypertension<br>• Pre-eclampsia<br>• Caesarean section<br>• Preterm delivery<br>• Small for gestational age

© 2021 Teede HJ et al. *JAMA Internal Medicine.*

Kennelly	2018	Ireland	535	29.3	Mixed	Standard care	**Primary**<br>• GDM<br>**Secondary**<br>• Gestational weight gain<br>• Physical activity<br>• Glycaemic index and load
Kiani Asiabar	2018	Iran	150	23.81	Mixed	Standard care	• Appropriate gestational weight gain (IOM guidelines)
Olson	2018	USA	1689	NR	Mixed	Standard care	**Primary**<br>• Exceeding upper limit of guidelines for total gestational weight gain<br>**Secondary**<br>• Excessive average weekly gestational weight gain in the last half of pregnancy<br>• Total gestational weight gain
Phelan	2018	USA	256	32.5	Diet with physical activity	At ~20-min welcome visit general information on healthy diet, physical activity, IOM guidelines on weight gain	**Primary**<br>• Gestational weight gain per week of observation<br>**Secondary**<br>• Proportions exceeding Institute of Medicine (IOM) guidelines for total gestational weight gain<br>• Changes in weight-control behaviors<br>• Cardiovascular disease risk factors<br>• Incidence of pregnancy complications
**Study**	**Publication Year**	**Country**	**Sample size**	**Body mass index**	**Intervention method**	**Comparison intervention**	**Outcomes**
Rönö	2018	Finland	492	32.15	Mixed	Standard care	**Primary**<br>• GDM<br>**Secondary**<br>• Achievement of dietary and physical activity goals<br>• Pregnancy-induced hypertension<br>• Pre-eclampsia<br>• Gestational weight gain<br>• Caesarean section<br>• Instrumental delivery<br>• Preterm delivery (<37 weeks)<br>• Large for gestational age
Al Wattar	2019	UK	1252	NR	Diet	Standard care	**Primary**<br>• Composite maternal (GDM or pre-eclampsia)

© 2021 Teede HJ et al. *JAMA Internal Medicine.*

Study	Publication Year	Country	Sample size	Body mass index	Intervention method	Comparison intervention	Outcomes
							• Composite neonate (stillbirth, small for gestational age, NICU)<br>**Secondary**<br>• Gestational weight gain<br>• GDM,<br>• Pre-eclampsia<br>• Preterm delivery (< 37 GW)<br>• Mode of delivery<br>• Maternal admission to high dependency/ intensive care<br>• Antepartum haemorrhage<br>• Maternal anaemia<br>• Perinatal death<br>• Small for gestational age<br>• Large for gestational age<br>• Admission to NICU
Anleu	2019	Chile	1002	NR	Diet	Standard care	• Total sugars consumption and energy
Barakat	2019	Spain	520	23.58	Physical activity	Standard care	**Primary**<br>• Gestational weight gain<br>• Excessive gestational weight gain (proportion over IOM)<br>• GDM<br>**Secondary**<br>• Gestational age at delivery
**Study**	**Publication Year**	**Country**	**Sample size**	**Body mass index**	**Intervention method**	**Comparison intervention**	**Outcomes**
							• Mode of delivery<br>• Birthweight<br>• Macrosomia (>4000 g)<br>• Low Birthweight (<2500 g)<br>Preterm delivery (< 37 GW)
Brik	2019	Spain	120	23.86	Physical activity	Advised not to attend any supervised exercise program for more than 30 min three times per week	**Primary**<br>• Gestational weight gain at 20, 28, 36 and 38 weeks<br>• Maternal weigh 6 weeks postpartum<br>**Secondary**<br>• Gestational age at delivery<br>• Caesarean section<br>• Preterm delivery (< 37 GW)<br>• Induction of labour<br>• Perinatal tear<br>• Birthweight

© 2021 Teede HJ et al. *JAMA Internal Medicine.*

							• 5-min Apgar score<6<br>• Arterial cord pH<br>• Admission to NICU<br>• Small for gestational age
Buckingham-Schutt	2019	USA	56	25	Diet with physical activity	Standard care	**Primary**<br>• Gestational weight gain<br>• Appropriate gestational weight gain (proportion within IOM-specific recommendations)<br>**Secondary**<br>• Pregnancy complications and foetal outcomes<br>• GDM<br>• Pregnancy-induced hypertension<br>• Pre-eclampsia<br>• Caesarean section<br>• Preterm delivery (< 37 GW)
Clark	2019	USA	42	26.34	Physical activity	No exercise intervention	**Primary**<br>• Offspring health outcomes<br>**Secondary**<br>• Gestational weight gain<br>• Total Cholesterol, HDL, Triglycerides<br>• Caesarean section
**Study**	**Publication Year**	**Country**	**Sample size**	**Body mass index**	**Intervention method**	**Comparison intervention**	**Outcomes**
Daley	2019	UK	616	26	Mixed	Standard care	**Primary**<br>• Excessive gestational weight gain (IOM guidelines)<br>**Secondary**<br>• Appropriate or inadequate gestational weight gain (IOM guidelines)<br>• Depression (changes baseline to 38 weeks)<br>• Anxiety (changes baseline to 38 weeks)<br>• Physical activity<br>• Diet quality<br>• Gestational weight gain<br>• GDM<br>• Pre-eclampsia<br>• Preterm delivery<br>• Perinatal mortality<br>• Admission to NICU

© 2021 Teede HJ et al. *JAMA Internal Medicine.*

Kunath	2019	Germany	2261	24.4	Mixed	Standard care	**<u>Primary</u>**<br>• Excessive gestational weight gain (proportion over IOM)<br>**<u>Secondary</u>**<br>• GDM<br>• Small for gestational age<br>• Large for gestational age<br>• Caesarean section
Okesene-Gafa	2019	New Zealand	230	38.56	Mixed	Standard care	**<u>Primary</u>**<br>• Excess weekly gestational weight gain (IOM guidelines)<br>• Small for gestational age<br>• Large for gestational age<br>**<u>Secondary</u>**<br>• Total gestational weight gain<br>• OGTT and Haemoglobin A1c at 28 and 36 weeks<br>• GDM<br>• Pregnancy-induced hypertension<br>• Caesarean section<br>• Depression and Anxiety<br>• Preterm delivery (<37 weeks)<br>• Perinatal mortality<br>• Admission to NICU
**Study**	**Publication Year**	**Country**	**Sample size**	**Body mass index**	**Intervention method**	**Comparison intervention**	**Outcomes**
Parat	2019	France	275	32.5	Mixed	Standard care	**<u>Primary</u>**<br>• Infant weight gain from birth to 2 years<br>**<u>Secondary</u>**<br>• Excess infant weight gain birth to 6 months and body mass index > 19 kg/m2 at 2 years (97th percentile)<br>• Large for gestational age<br>• Exclusive breastfeeding at discharge and 4 months<br>• GDM<br>• Pregnancy-induced hypertension<br>• Pre-eclampsia<br>• Caesarean section<br>• Instrumental delivery<br>• Maternal body mass index 1 and 2 years after delivery<br>• Diabetes 2 years after delivery<br>• Maternal quality of life 2 years after delivery<br>• Perinatal mortality<br>• Small for gestational age

© 2021 Teede HJ et al. *JAMA Internal Medicine.*

							• Large for gestational age
Pelaez	2019	Spain	345	23.7	Physical activity	Standard care	**Primary**<br>• Gestational weight gain<br>**Secondary**<br>• GDM<br>• Macrosomia<br>• Caesarean section<br>• Instrumental delivery
Arthur	2020	Australia	396	27.49	Mixed	Standard care	**Primary**<br>• Percentage weight change above target range<br>**Secondary**<br>• Change in weight (kg/week)<br>• Proportion with weight gain over IOM guidelines<br>• Gestational weight gain<br>• GDM<br>• Pregnancy-induced hypertension<br>• Pre-eclampsia<br>• Caesarean section<br>• Admission to NICU
**Study**	**Publication Year**	**Country**	**Sample size**	**Body mass index**	**Intervention method**	**Comparison intervention**	**Outcomes**
Ferrara	2020	USA	398	29.40	Diet with Physical activity	Standard care	**Primary**<br>• Weekly rate of gestational weight gain<br>**Secondary**<br>• Total gestational weight gain<br>• Excess gestational weight gain (IOM guidelines)<br>• Proportion meeting lower limit gestational weight gain<br>• Changes in total caloric intake during pregnancy<br>• Changes in proportion of calories from total fat and saturated and unsaturated fat<br>• Changes in physical activity<br>• Changes in serum metabolic markers<br>• Small for gestational age<br>• Large for gestational age<br>• Macrosomia<br>• Low birthweight<br>• Pregnancy loss<br>• Preterm delivery (<37 weeks)<br>• Caesarean section<br>• Pregnancy-induced hypertension and Pre-eclampsia

© 2021 Teede HJ et al. *JAMA Internal Medicine.*

							• GDM
Rodríguez-Blanque	2020	Spain	162	24.41	Physical activity	Standard care	• Mode of delivery (proportion delivery modes by normal and overweight/obesity)<br>• Gestational weight gain
Trak-Fellermeier [88]	2020	USA	31	35.3	Diet with Physical activity	No intervention	**Primary**<br>• Weekly gestational weight gain (proportion inside and outside of IOM guidelines)<br>**Secondary**<br>• Gestational weight gain (proportion inside and outside of IOM guidelines)<br>• Small for gestational age<br>• Large for gestational age<br>• GDM<br>• Caesarean section<br>• Pre-eclampsia<br>• Preterm delivery (<37 weeks)<br>• Admission to NICU

GDM= Gestational diabetes, NICU= Neonatal intensive care unit, IGF =insulin like growth factor

© 2021 Teede HJ et al. *JAMA Internal Medicine.*

eReferences

1. Gomez Tabares G, Delgado JG, Agudelo AA, Hurtado H. Efecto De La Dieta En El Resultado Perinatal De La Paciente Obesa Embarazada. *Review Colombian Obstetrics and Ginecology.* 1994;45(4):313-316
2. Lee G. Exercise in Pregnancy. *Modern Midwife*. 1996;6(8):28-33.
3. Kihlstrand M, Stenman B, Nilsson S, Axelsson O. Water-gymnastics reduced the intensity of back/low back pain in pregnant women. *Acta Obstetrica Gynecologicia Scandanavia*. 1999;78(3):180-185.
4. Bechtel-Blackwell DA. Computer-Assisted Self-Interview and Nutrition Education in Pregnant Teens. *Clinical Nursing Research* 2002;11(4):450-462.
5. Briley C, Flanagan NL, Lewis NM. In-home prenatal nutrition intervention increased dietary iron intakes and reduced low birthweight in low-income African-American women. *Journal of American Dietetics Association*. 2002;102(7):984-987.
6. Clapp JF, Kim H, Burciu B, Lopez B. Beginning regular exercise in early pregnancy: Effect on fetoplacental growth. *American Journal Obstetrics Gynecology*. 2000;183(6):1484-1488.
7. Marquez-Sterling S, Perry AC, Kaplan TA, Halberstein RA, Signorile JF. Physical and psychological changes with vigorous exercise in sedentary primigravidae. *Medicine Science Sports Exercise*. 2000;32(1):58-62.
8. Polley BA, Wing RR, Sims CJ. Randomized controlled trial to prevent excessive weight gain in pregnant women. *Inernational Journal of Obesity*. 2002;26(11):1494-1502.
9. Prevedel TTS, Calderon I de MP, De Conti MH, Consonni EB, Rudge MVC. Repercussões maternas e perinatais da hidroterapia na gravidez. *Brazilian Journal of Gynecology and Obstetrics*. 2003;25(1):53-59.
10. Garshasbi A, Faghih Zadeh S. The effect of exercise on the intensity of low back pain in pregnant women. *International Journal Gynaecology and Obstetrics* 2005;88(3):271-275.
11. Khoury J, Henriksen T, Christophersen B, Tonstad S. Effect of a cholesterol-lowering diet on maternal, cord, and neonatal lipids, and pregnancy outcome: A randomized clinical trial. *American Journal Obstetrics and Gynecology*. 2005;193(4):1292-1301
12. Santos IA, Stein R, Fuchs SC, et al. Aerobic exercise and submaximal functional capacity in overweight pregnant women: A randomized trial. *Obstetrics and Gynecology*. 2005;106(2):243-249.
13. Sedaghati P, Ziaee V, Ardjmand A. The effect of an ergometric training program on pregnants weight gain and low back pain. Gazzetta Medica Italiana 2007; 166(6):209-13
14. Baciuk EP, Pereira RI, Cecatti JG, Braga AF, Cavalcante SR. Water aerobics in pregnancy: cardiovascular response, labor and neonatal outcomes. *Reproductive Health*. 2008;5:10.
15. Barakat R, Stirling JR, Lucia A. Does exercise training during pregnancy affect gestational age? A randomised controlled trial. *British Journal Sports Medicine*. 2008;42(8):674-678.
16. Wolff S, Legarth J, Vangsgaard K, Toubro S, Astrup A. A randomized trial of the effects of dietary counseling on gestational weight gain and glucose metabolism in obese pregnant women. *International Journal Obesity*. 2008;32:495-501.
17. Asbee SM, Jenkins TR, Butler JR, White J, Elliot M, Rutledge A. Preventing excessive weight gain during pregnancy through dietary and lifestyle counseling: a randomized controlled trial. *Obstetrics Gynecology*. 2009;113(2 Pt 1):305-312.
18. Jeffries K, Shub A, Walker SP, Hiscock R, Permezel M. Reducing excessive weight gain in pregnancy: A randomised controlled trial. *Medical Journal Australia* 2009;191(8):429-433.
19. Ong MJ, Guelfi KJ, Hunter T, Wallman KE, Fournier PA, Newnham JP. Supervised home-based exercise may attenuate the decline of glucose tolerance in obese pregnant women. *Diabetes Metabolism*. 2009;35(5):418-421.
20. Thornton YS, Smarkola C, Kopacz SM, Ishoof SB. Perinatal outcomes in nutritionally monitored obese pregnant women: A randomized clinical trial. *Journal National Medical Association*. 2009;101(6):569-577.
21. Guelinckx I, Devlieger R, Mullie P, Vansant G. Effect of lifestyle intervention on dietary habits, physical activity, and gestational weight gain in obese pregnant women: A randomized controlled trial. *American Journal Clinical Nutrition*. 2010;91(2):373-380.
22. Hopkins SA, Baldi JC, Cutfield WS, McCowan L, Hofman PL. Exercise Training in Pregnancy Reduces Offspring Size without Changes in Maternal Insulin Sensitivity. *Journal Clinical Endocrinology Metabolism*. 2010;95(5):2080-2088.
23. Khaledan A, Mirdar S, Motahari Tabari NS, Ahmad Shirvani M. Effect of an Aerobic Exercise Program on Fetal Growth in Pregnant Women. *Journal of Hayat*. 2010;16(1):55-64.
24. Barakat R, Pelaez M, Montejo R, Luaces M, Zakynthinaki M. Exercise during pregnancy improves maternal health perception: A randomized controlled trial. *American Journal Obstetrics Gynecology*. 2011;204(5):402.e1-402.e7.
25. Haakstad LAH, Bø K. Effect of regular exercise on prevention of excessive weight gain in pregnancy: A randomised controlled trial. *Euroepan Journal Contraception Reproductie Health Care*. 2011;16(2):116-125.
26. Huang TT, Yeh CY, Tsai YC. A diet and physical activity intervention for preventing weight retention among Taiwanese childbearing women: a randomised controlled trial. *Midwifery*. 2011;27(2):257-264.

© 2021 Teede HJ et al. *JAMA Internal Medicine.*

27. Jackson RA, Stotland NE, Caughey AB, Gerbert B. Improving diet and exercise in pregnancy with Video Doctor counseling: a randomized trial. *Patient Education Counselling*. 2011;83(2):203-209.
28. Nascimento SL, Surita FG, Parpinelli MÂ, Siani S, Pinto e Silva JL. The effect of an antenatal physical exercise programme on maternal/perinatal outcomes and quality of life in overweight and obese pregnant women: a randomised clinical trial. *BJOG An International Journal Obstetrics Gynaecology*. 2011;118(12):1455-1463.
29. Phelan S, Phipps MG, Abrams B, Darroch F, Schaffner A, Wing RR. Randomized trial of a behavioral intervention to prevent excessive gestational weight gain: the Fit for Delivery Study. *American Journal Clinical Nutrition*. 2011;93:772–9.
30. Quinlivan JA, Lam LT, Fisher J. A randomised trial of a four-step multidisciplinary approach to the antenatal care of obese pregnant women. *Australian New Zealeand Journal Obstetrics Gynaecologhy*. 2011;51(2):141-146.
31. Vinter CA, Jensen DM, Ovesen P, Beck-Nielsen H, Jørgensen JS. The LiP (Lifestyle in Pregnancy) study: A randomized controlled trial of lifestyle intervention in 360 obese pregnant women. *Diabetes Care*. 2011;34(12):2502-2507.
32. Barakat R, Pelaez M, Lopez C, Montejo R, Coteron J. Exercise during pregnancy reduces the rate of cesarean and instrumental deliveries: Results of a randomized controlled trial. *Journal Maternal Neonatal Medicine*. 2012;25(11):2372-2376.
33. Barakat R, Cordero Y, Coteron J, Luaces M, Montejo R. Exercise during pregnancy improves maternal glucose screen at 24-28 weeks: A randomised controlled trial. *British Journal Sports Medicine*. 2012;46(9):656-661.
34. de Oliveria Melo AS, Silva JLP, Tavares JS, Barros VO, Leite DFB, Amorim MMR. Effect of a physical exercise program during pregnancy on uteroplacental and fetal blood flow and fetal growth: A randomized controlled trial. *Obstetrics Gynecology*. 2012;120(2):302-310.
35. Hui A, Back L, Ludwig S, et al. Lifestyle intervention on diet and exercise reduced excessive gestational weight gain in pregnant women under a randomised controlled trial. *BJOG An International Journal Obstetrics Gynaecology*. 2012;119(1):70-77.
36. Korpi-Hyövälti E, Heinonen S, Schwab U, Laaksonen DE, Niskanen L. Effect of intensive counselling on physical activity in pregnant women at high risk for gestational diabetes mellitus. A clinical study in primary care. *Primary Care Diabetes*. 2012;6(4):261-268.
37. Oostdam N, Van Poppel MNM, Wouters MGAJ, et al. No effect of the FitFor2 exercise programme on blood glucose, insulin sensitivity, and birthweight in pregnant women who were overweight and at risk for gestational diabetes: Results of a randomised controlled trial. *BJOG An International Journal Obstetrics Gynaecology*. 2012;119(9):1098-1107.
38. Price B, Amini S, Kapperler K. Exercise in Pregnancy: Effect on Fitness and Obstetric Outcomes—A Randomized Trial. *Medicine Science Sport*. 2012;44(12):2263–2269.
39. Rakhshani A, Nagarathna R, Mhaskar R, Mhaskar A, Thomas A, Gunasheela S. The effects of yoga in prevention of pregnancy complications in high-risk pregnancies: A randomized controlled trial. *Preventive Medicine (Baltim)*. 2012;55(4):333-340.
40. Ramírez-Vélez R, Lobelo F, Aguilar-de Plata AC, Izquierdo M, García-Hermoso A. Exercise during pregnancy on maternal lipids: A secondary analysis of randomized controlled trial. *BMC Pregnancy Childbirth*. 2017;17(1):1-9.
41. Stafne SN, Salvesen KÅ, Romundstad PR, Torjusen IH, Mørkved S. Does regular exercise including pelvic floor muscle training prevent urinary and anal incontinence during pregnancy? A randomised controlled trial. *BJOG An International Journal Obstetrics Gynaecology*. 2012;119(10):1270-1280.
42. Walsh JM, McGowan CA, Mahony R, Foley ME, McAuliffe FM. Low glycaemic index diet in pregnancy to prevent macrosomia (ROLO study): Randomised control trial. *British Medical Journal*. 2012;345(7875):1-9.
43. Althuizen E, Van Der Wijden CL, Van Mechelen W, Seidell JC, Van Poppel MNM. The effect of a counselling intervention on weight changes during and after pregnancy: A randomised trial. *BJOG An Intrnational Journal Obstetrics Gynaecology*. 2013;120(1):92-99.
44. Barakat R, Pelaez M, Lopez C, Lucia A, Ruiz JR. Exercise during pregnancy and gestational diabetes-related adverse effects: a randomised controlled trial. *British Journal Sports Medicine*. 2013;47(10):630-636.
45. Bogaerts AFL, Devlieger R, Nuyts E, Witters I, Gyselaers W, Van Den Bergh BRH. Effects of lifestyle intervention in obese pregnant women on gestational weight gain and mental health: A randomized controlled trial. *International Journal Obes*ity. 2013;37(6):814-821.
46. Deveer R, Deveer M, Akbaba E, et al. The effect of diet on pregnancy outcomes among pregnants with abnormal glucose challenge test. *European Review Medical Pharmacolical Sciences*. 2013;17(9):1258-1261.
47. Ruiz J, Perales M, Pelaez M, Lopez C, Lucia A, Barakat R. Supervised Exercise-Based Intervention to Prevent Excessive. *Mayo Clinical Proceedings*. 2013;88(12):1388-1397.
48. Tomić V, Sporiš G, Tomić J, Milanović Z, Zigmundovac-Klaić D, Pantelić S. The effect of maternal exercise during pregnancy on abnormal fetal growth. *Croatian Medical Journal*. 2013;54(4):362-368.
49. Barakat R, Perales M, Bacchi M, Coteron J, Refoyo I. A program of exercise throughout pregnancy. is it safe to mother and newborn? *American Journal Health Promotion*. 2014;29(1):2-8.

© 2021 Teede HJ et al. *JAMA Internal Medicine.*

50. Di Carlo C, Iannotti G, Sparice S, et al. The role of a personalized dietary intervention in managing gestational weight gain: a prospective, controlled study in a low-risk antenatal population. *Archives Gynecology Obstetrics*. 2014;289(4):765-770.
51. Dodd JM, Turnbull D, McPhee AJ, et al. Antenatal lifestyle advice for women who are overweight or obese: LIMIT randomised trial. *British Medical Journal*. 2014;348(February):5-7.
52. Hui AL, Back L, Ludwig S, et al. Effects of lifestyle intervention on dietary intake, physical activity level, and gestational weight gain in pregnant women with different pre-pregnancy Body Mass Index in a randomized control trial. *BMC Pregnancy Childbirth*. 2014;14(1):1-9.
53. Ko CW, Napolitano PG, Lee SP, Schulte SD, Ciol MA, Beresford SAA. Physical activity, maternal metabolic measures, and the incidence of gallbladder sludge or stones during pregnancy: A randomized trial. *American Journal Perinatology*. 2014;31(1):39-48.
54. Kong KL, Campbell CG, Foster RC, Peterson AD, Lanningham-Foster L. A pilot walking program promotes moderate-intensity physical activity during pregnancy. *Medicne Science Sports Exercise*. 2014;46(3):462-471.
55. Li Q, Cui H, Zheng D, Li N, Chang L, Liu C. Effects of walking exercise during late trimester on pregnancy outcome of low-risk primipara. *Zhonghua Yi Xue Za Zhi*. 2014;94(22):1722-1725.
56. Petrella E, Malavolti M, Bertarini V, et al. Gestational weight gain in overweight and obese women enrolled in a healthy lifestyle and eating habits program. *Journal Maternal Neonatal Medicine*. 2014;27(13):1348-1352.
57. Renault KM, Nørgaard K, Nilas L, et al. The Treatment of Obese Pregnant Women (TOP) study: A randomized controlled trial of the effect of physical activity intervention assessed by pedometer with or without dietary intervention in obese pregnant women. *American Journal Obstetrics Gynecoogyl*. 2014;210(2):134.e1-134.e9.
58. Vesco KK, Karanja N, King JC, et al. Efficacy of a group-based dietary intervention for limiting gestational weight gain among obese women: A randomized trial. *Obesity*. 2014;22(9):1989-1996.
59. Bisson M, Alméras N, Dufresne SS, et al. A 12-week exercise program for pregnant women with obesity to improve physical activity levels: An open randomised preliminary study. *PLoS One*. 2015;10(9):1-17.
60. Cordero Y, Mottola MF, Vargas J, Blanco M, Barakat R. Exercise is associated with a reduction in gestational diabetes mellitus. *Med Sci Sports Exerc*. 2015;47(7):1328-1333.
61. Dekker Nitert M, Barrett HL, Denny KJ, McIntyre HD, Callaway LK. Exercise in pregnancy does not alter gestational weight gain, MCP-1 or leptin in obese women. *Australian New Zealand Journal Obstetrics Gynaecology*. 2015;55(1):27-33.
62. Gesell SB, Katula JA, Strickland C, Vitolins MZ. Feasibility and Initial Efficacy Evaluation of a Community-Based Cognitive-Behavioral Lifestyle Intervention to Prevent Excessive Weight Gain During Pregnancy in Latina Women. *Maternal Child Health Journal*. 2015;19(8):1842-1852.
63. Hawkins M, Hosker M, Marcus BH, et al. A pregnancy lifestyle intervention to prevent gestational diabetes risk factors in overweight Hispanic women: a feasibility randomized controlled trial. *Diabetic Medicine*. 2015;32(1):108-115.
64. Jing W, Huang Y, Liu X, Luo B, Yang Y, Liao S. The effect of a personalized intervention on weight gain and physical activity among pregnant women in China. *International Journal Gynecology Obstetrics*. 2015;129(2):138-141.
65. Perales M, Refoyo I, Coteron J, Bacchi M, Barakat R. Exercise During Pregnancy Attenuates Prenatal Depression: A Randomized Controlled Trial. *Evaluation Heathl Professional*. 2015;38(1):59-72.
66. Petrov Fieril K, Glantz A, Fagevik Olsen M. The efficacy of moderate-to-vigorous resistance exercise during pregnancy: A randomized controlled trial. *Acta Obstetrica Gynecology Scandanavia*. 2015;94(1):35-42.
67. Poston L, Bell R, Croker H, et al. Effect of a behavioural intervention in obese pregnant women (the UPBEAT study): A multicentre, randomised controlled trial. *Lancet Diabetes Endocrinology*. 2015;3(10):767-777.
68. Ronnberg AK, Ostlund I, Fadl H, Gottvall T, Nilsson K. Intervention during pregnancy to reduce excessive gestational weight gain—a randomised controlled trial. *BJOG An International Journal Obstetrics Gynaecology*. 2015;122(4):537-544.
69. Aşcı Ö, Rathfisch G. Effect of lifestyle interventions of pregnant women on their dietary habits, lifestyle behaviors, and weight gain: a randomized controlled trial. *Journal Health Population Nutrition*. 2016;35:7.
70. Barakat R, Pelaez M, Cordero Y, et al. Exercise during pregnancy protects against hypertension and macrosomia: Randomized clinical trial. *American Journal Obstetrics Gynecology* 2016;214(5):649.
71. Garnæs KK, Mørkved S, Salvesen Ø, Moholdt T. Exercise Training and Weight Gain in Obese Pregnant Women: A Randomized Controlled Trial (ETIP Trial). *PLoS Medicine*. 2016;13(7):e1002079.
72. Herring SJ, Cruice JF, Bennett GG, Rose MZ, Davey A, Foster GD. Preventing excessive gestational weight gain among African American women: A randomized clinical trial. *Obesity*. 2016;24(1):30-36.
73. Koivusalo SB, Rönö K, Klemetti MM, et al. Gestational Diabetes Mellitus Can Be Prevented by Lifestyle Intervention: The Finnish Gestational Diabetes Prevention Study (RADIEL): A Randomized Controlled Trial. *Diabetes Care*. 2016;39(1):24-30.
74. McCarthy EA, Walker SP, Ugoni A, Lappas M, Leong O, Shub A. Self-weighing and simple dietary advice for overweight and obese pregnant women to reduce obstetric complications without impact on quality of life: A

© 2021 Teede HJ et al. *JAMA Internal Medicine.*

randomised controlled trial. *BJOG An International Journal Obstetrics Gynaecology*. 2016;123(6):965-973. 75. Perales M, Calabria I, Lopez C, Franco E, Coteron J, Barakat R. Regular Exercise Throughout Pregnancy is Associated with a Shorter First Stage of Labor. *American Journal Health Promotion*. 2016;30(3):149-154.

75. Perales M, Santos-Lozano A, Sanchis-Gomar F, et al. Maternal cardiac adaptations to a physical exercise program during pregnancy. *Medicine Science Sports Exercise*. 2016;48(5):896-906.

77. Seneviratne SN, Jiang Y, Derraik JGB, et al. Effects of antenatal exercise in overweight and obese pregnant women on maternal and perinatal outcomes: A randomised controlled trial. *BJOG An International Journal Obstetrics Gynaecology*. 2016;123(4):588-597.

78. Smith K, Lanningham-Foster L, Welch A, Campbell C. Web-Based Behavioral Intervention Increases Maternal Exercise but Does Not Prevent Excessive Gestational Weight Gain in Previously Sedentary Women. *Journal Physical Activity Health*. 2016;13(6):587-593.

79. Sun Y, Zhao H. The effectiveness of lifestyle intervention in early pregnancy to prevent gestational diabetes mellitus in Chinese overweight and obese women: A quasi-experimental study. *Applied Nursing Research*. 2016;30:125-130.

80. Toosi M, Akbarzadeh M. The Effect of Aerobic Exercises on Maternal Outcomes: A Randomized Controlled Clinical Trial. *Women's Health Bulletin*. 2016;3(4).

81. Wang C, Wei Y, Zhang X, et al. Effect of regular exercise commenced in early pregnancy on the incidence of gestational diabetes mellitus in overweight and obese pregnant women: A randomized controlled trial. *Diabetes Care*. 2016;39(10):e163-e164.

82. Bruno R, Petrella E, Bertarini V, Pedrielli G, Neri I, Facchinetti F. Adherence to a lifestyle programme in overweight/obese pregnant women and effect on gestational diabetes mellitus: a randomized controlled trial. *Maternl Child Nutrition*. 2017;13(3):1-11.

83. Daly N, Farren M, McKeating A, O'Higgins A, Mullaney L, Turner MJ. 34: Effect of a randomized controlled trial of an intensive medically supervised exercise program designed to improve maternal glucose control on gestational weight gain. *Ameriana Journal Obstetrics Gynecology*. 2017;216(1, Supplement):S24.

84. Van Horn L, Peaceman A, Kwasny M, et al. Dietary Approaches to Stop Hypertension Diet and Activity to Limit Gestational Weight: Maternal Offspring Metabolics Family Intervention Trial, a Technology Enhanced Randomized Trial. *American Journal Preventive Medicine*. 2018;55(5):603-614.

85. Sagedal LR, Øverby NC, Bere E, et al. Lifestyle intervention to limit gestational weight gain: the Norwegian Fit for Delivery randomised controlled trial. *BJOG An International Journal Obstetrics Gynaecology*. 2017;124(1):97-109.

86. Simmons D, Devlieger R, Van Assche A, et al. Effect of physical activity and/or healthy eating ongdm risk: The dali lifestyle study. *Journal Clinical Endocrinology Metabolism*. 2017;102(3):903-913.

87. Willcox JC, Wilkinson SA, Lappas M, et al. A mobile health intervention promoting healthy gestational weight gain for women entering pregnancy at a high body mass index: the txt4two pilot randomised controlled trial. *BJOG An International Journal Obstetrics Gynaecology*. 2017;124(11):1718-1728.

88. Trak-Fellermeier MA, Campos M, Meléndez M, et al. Pearls randomized lifestyle trial in pregnant hispanic women with overweight/obesity: Gestational weight gain and offspring birthweight. *Diabetes, Metabolism Syndrome Obesity Targets Therapeutics*. 2019;12:225-238.

© 2021 Teede HJ et al. *JAMA Internal Medicine.*

eFigure 1. Funnel Plots and Egger's Tests Exploring Potential Publication Bias; Gestational Weight Gain (panel A), Maternal and Neonatal Outcomes Across Gestational Diabetes, Hypertensive Disorders of Pregnancy, Preterm Delivery, Cesarean Section, Fetal Death, Small for Gestational Age, Large for Gestational Age and Neonatal Intensive Care admission (panels B-I)

A) Funnel plot for Gestational Weight Gain (n=99 studies)

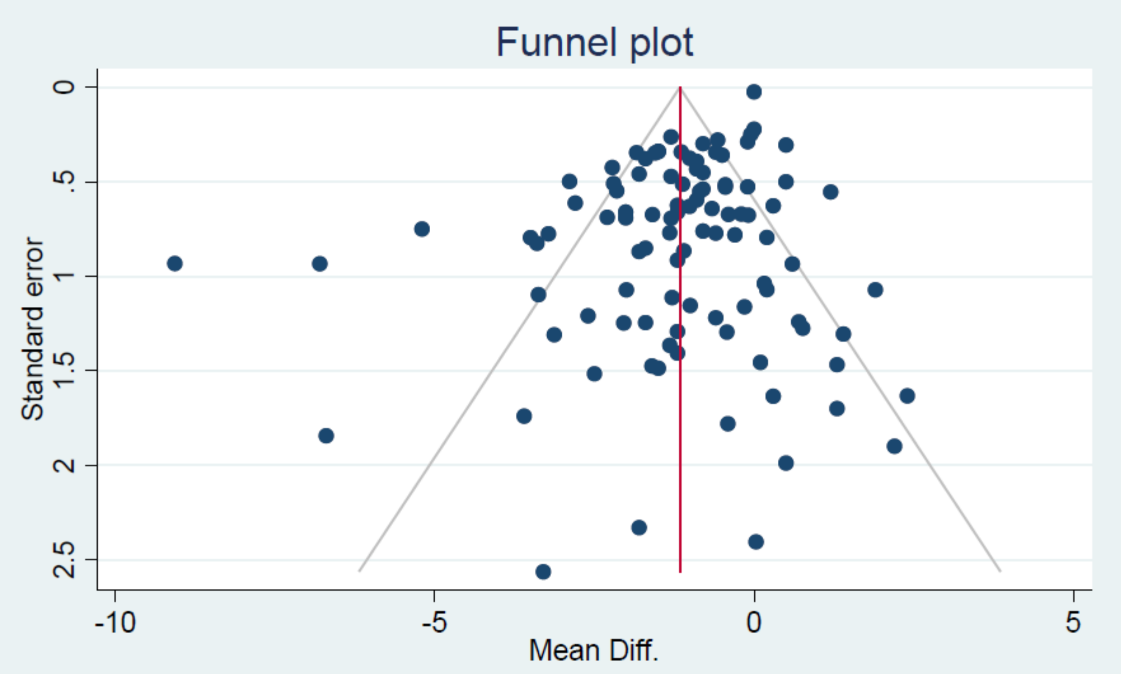

Egger's test for small-study effects: Regress standard normal deviate of intervention effect estimate against its standard error

Number of studies = 80 Root MSE = 8.212

Std_Eff	Coef.	Std. Err.	t	P>|t|	[95% Conf. Interval]	
slope	-5.427586	.4714589	-11.51	0.000	-6.366188	-4.488983
bias	-9.507576	1.744395	-5.45	0.000	-12.9804	-6.034753

Test of H0: no small-study effects P = 0.000

© 2021 Teede HJ et al. *JAMA Internal Medicine.*

B) Funnel plot for GDM (n=67 studies)

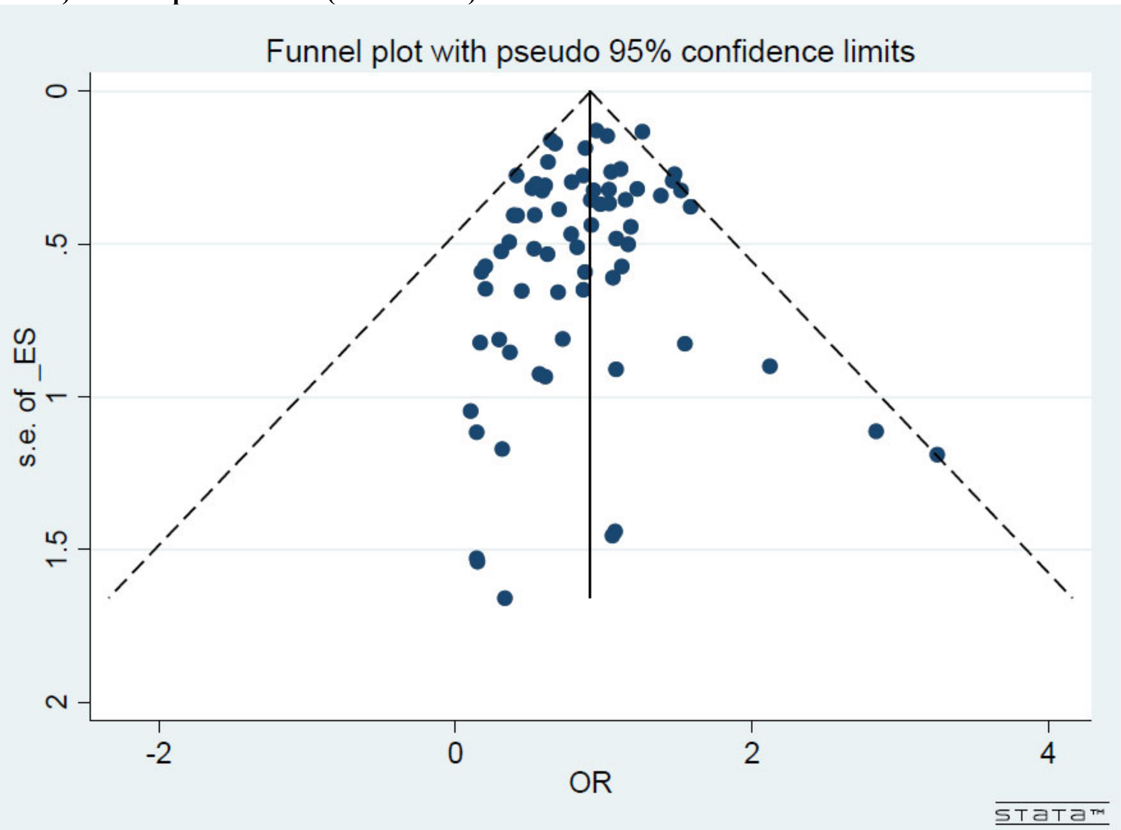

Egger's test for small-study effects: Regress standard normal deviate of intervention effect estimate against its standard error

Number of studies = 67 Root MSE = .9976

Std_Eff	Coef.	Std. Err.	t	P>|t|	[95% Conf. Interval]	
slope	.9880208	.0742558	13.31	0.000	.8398458	1.136196
bias	-.2722564	.2206908	-1.23	0.222	-.712638	.1681252

Test of H0: no small-study effects P = 0.222

© 2021 Teede HJ et al. *JAMA Internal Medicine.*

C) Funnel plot for Hypertensive disorders of pregnancy (n=53 studies)

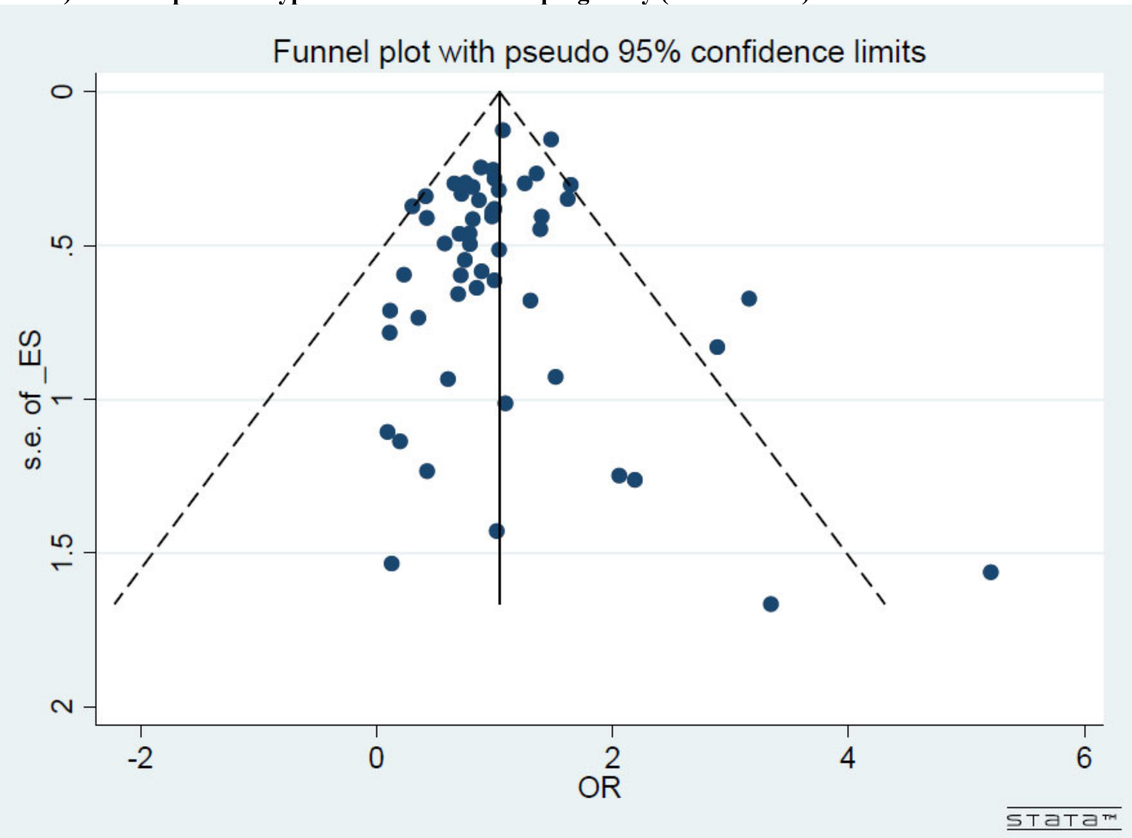

Egger's test for small-study effects: Regress standard normal deviate of intervention effect estimate against its standard error

Number of studies = 53 Root MSE = 1.155

Std_Eff	Coef.	Std. Err.	t	P>|t|	[95% Conf. Interval]	
slope	1.099812	.1135184	9.69	0.000	.8720208	1.327603
bias	-.1725028	.2999886	-0.58	0.568	-.7744739	.4294683

Test of H0: no small-study effects P = 0.568

© 2021 Teede HJ et al. *JAMA Internal Medicine.*

D) Funnel plot for Preterm delivery (n=52 studies)

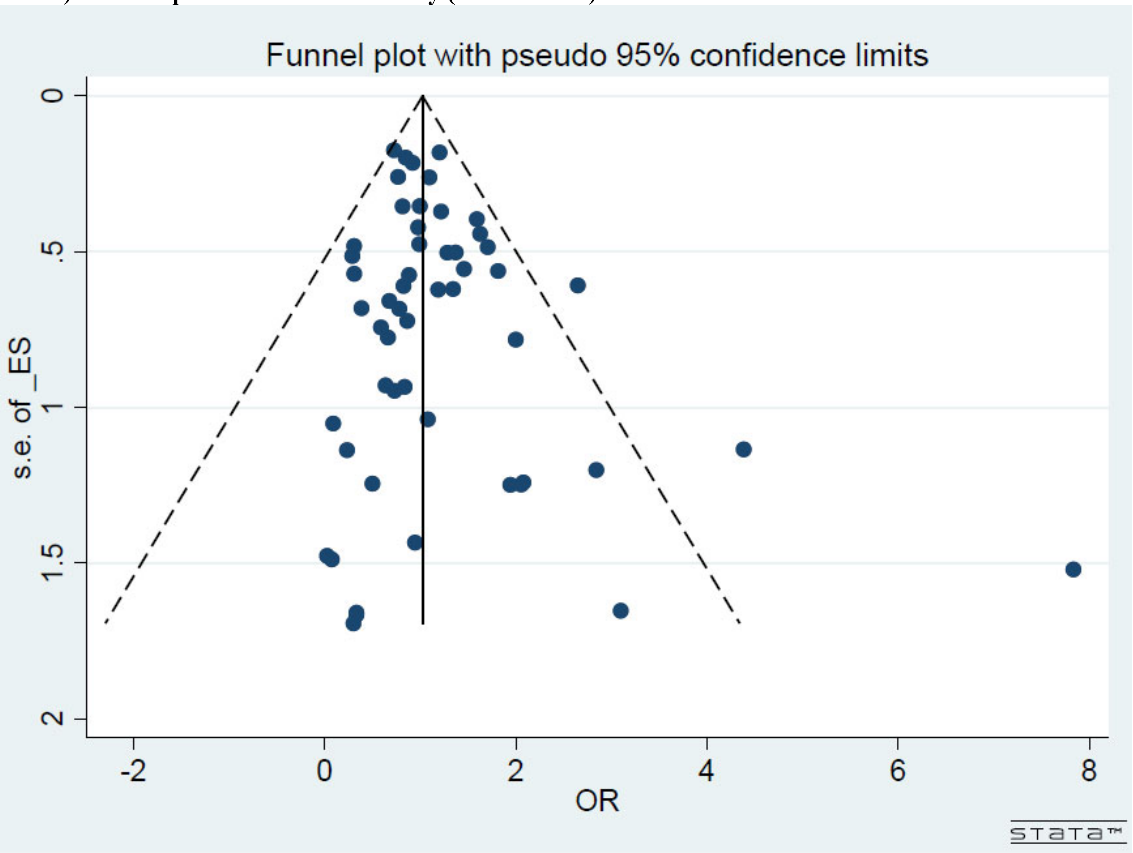

Egger's test for small-study effects: Regress standard normal deviate of intervention effect estimate against its standard error

Number of studies = 52 Root MSE = 1.142

Std_Eff	Coef.	Std. Err.	t	P>|t|	[95% Conf. Interval]	
slope	.8405569	.1253309	6.71	0.000	.5889446	1.092169
bias	.4915156	.2755363	1.78	0.080	-.0616467	1.044678

Test of H0: no small-study effects P = 0.080

© 2021 Teede HJ et al. *JAMA Internal Medicine.*

E) Funnel plot for Caesarean section (n=76 studies)

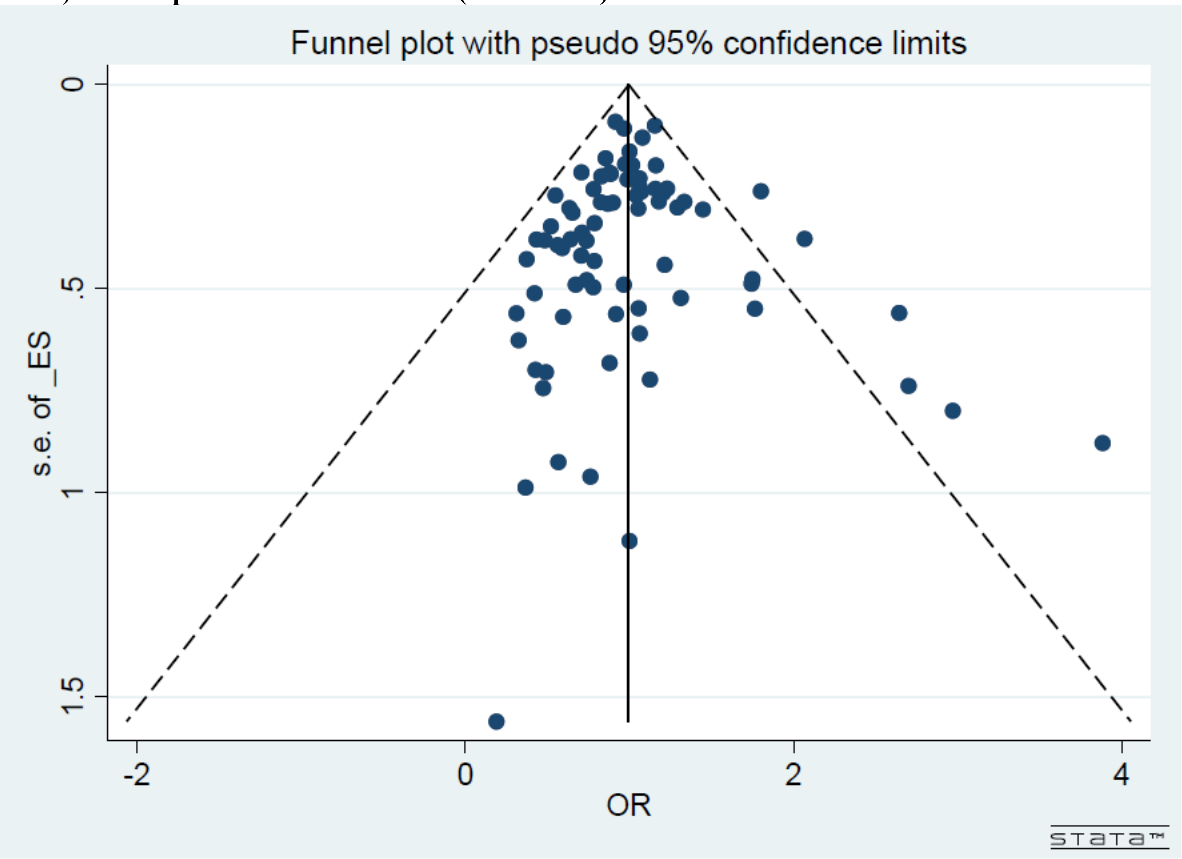

Egger's test for small-study effects: Regress standard normal deviate of intervention effect estimate against its standard error

Number of studies = 76 Root MSE = 1.15

Std_Eff	Coef.	Std. Err.	t	P>|t|	[95% Conf. Interval]	
slope	.9903931	.0671271	14.75	0.000	.8566692	1.124117
bias	.0222706	.2486227	0.09	0.929	-.4730111	.5175524

Test of H0: no small-study effects P = 0.929

© 2021 Teede HJ et al. *JAMA Internal Medicine.*

F) Funnel plot for Fetal death (n=12 studies)

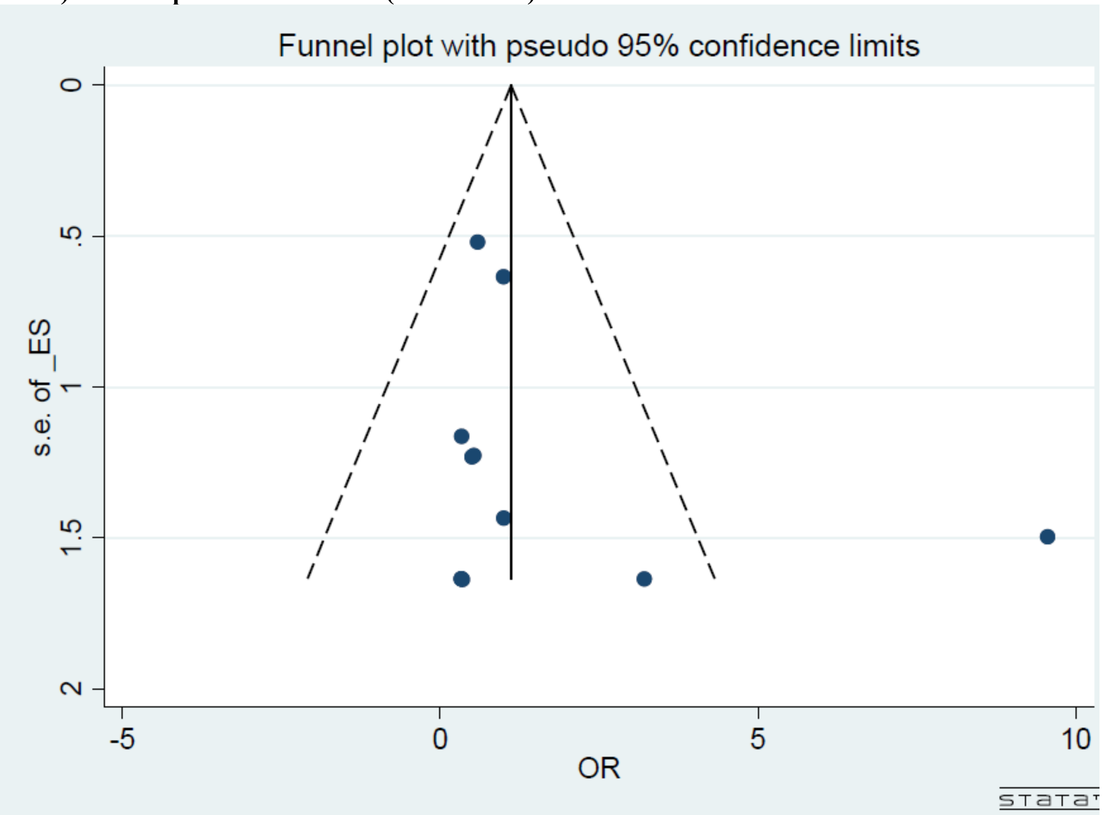

Egger's test for small-study effects: Regress standard normal deviate of intervention effect estimate against its standard error

Number of studies = 11 Root MSE = 1.913

Std_Eff	Coef.	Std. Err.	t	P>|t|	[95% Conf. Interval]	
slope	-.0522351	1.367137	-0.04	0.970	-3.144913	3.040443
bias	1.274404	1.348111	0.95	0.369	-1.775235	4.324043

Test of H0: no small-study effects P = 0.369

© 2021 Teede HJ et al. *JAMA Internal Medicine.*

G) Funnel plot for Small for gestational age (n=24 studies)

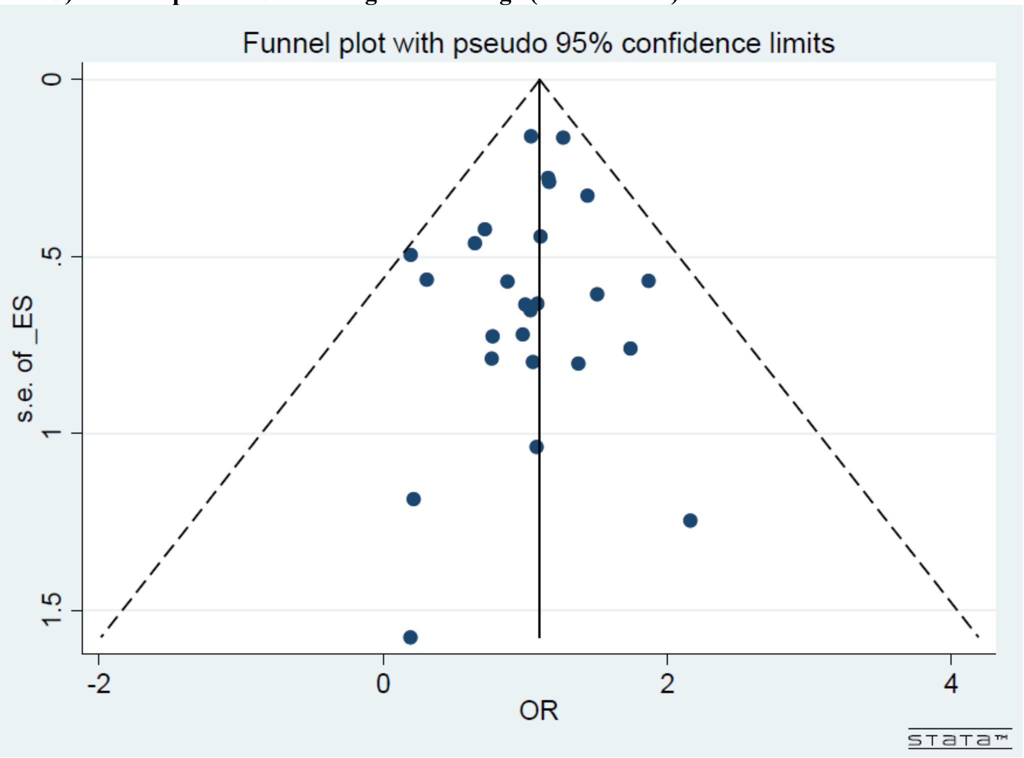

Egger's test for small-study effects: Regress standard normal deviate of intervention effect estimate against its standard error

Number of studies = 24 Root MSE = .7702

Std_Eff	Coef.	Std. Err.	t	P>|t|	[95% Conf. Interval]	
slope	1.190578	.1082619	11.00	0.000	.9671361	1.414019
bias	-.2664412	.2711048	-0.98	0.336	-.825974	.2930915

Test of H0: no small-study effects P = 0.336

© 2021 Teede HJ et al. *JAMA Internal Medicine.*

H) Funnel plot for Large for gestational age (n=28 studies)

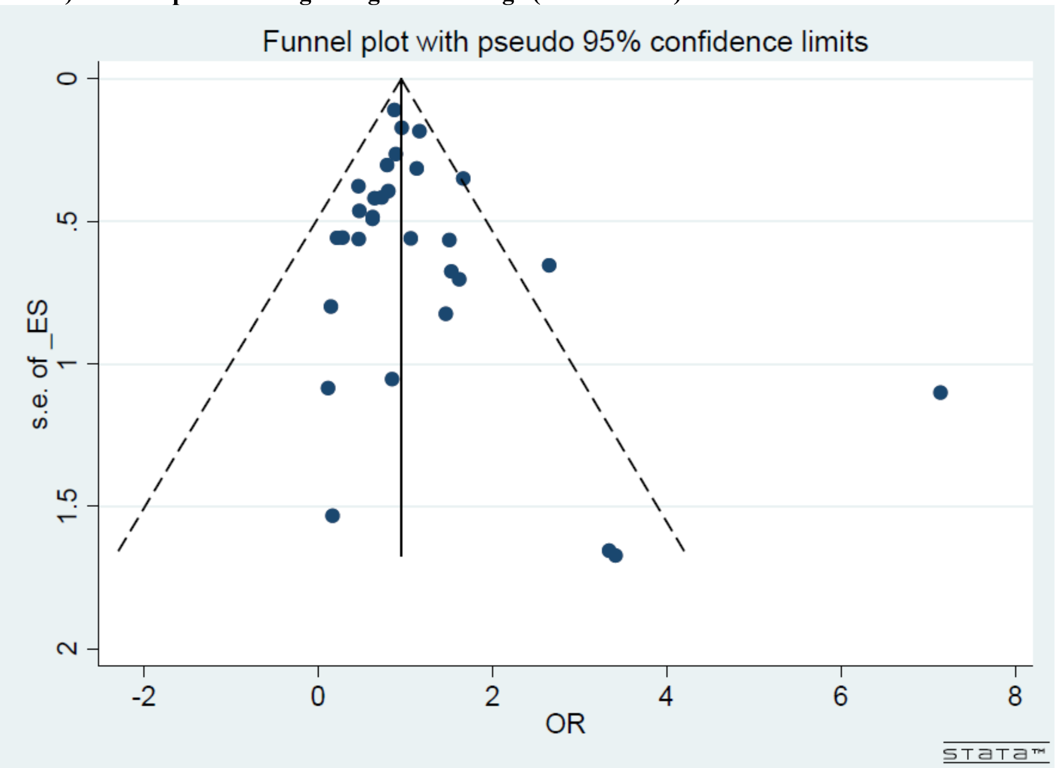

Egger's test for small-study effects: Regress standard normal deviate of intervention effect estimate against its standard error

Number of studies = 28 Root MSE = 1.46

Std_Eff	Coef.	Std. Err.	t	P>|t|	[95% Conf. Interval]	
slope	.8101572	.1495729	5.42	0.000	.5037709	1.116543
bias	.5355413	.4410383	1.21	0.235	-.3678847	1.438967

Test of H0: no small-study effects P = 0.235

© 2021 Teede HJ et al. *JAMA Internal Medicine.*

I) Funnel plot for NICU (n=17 studies)

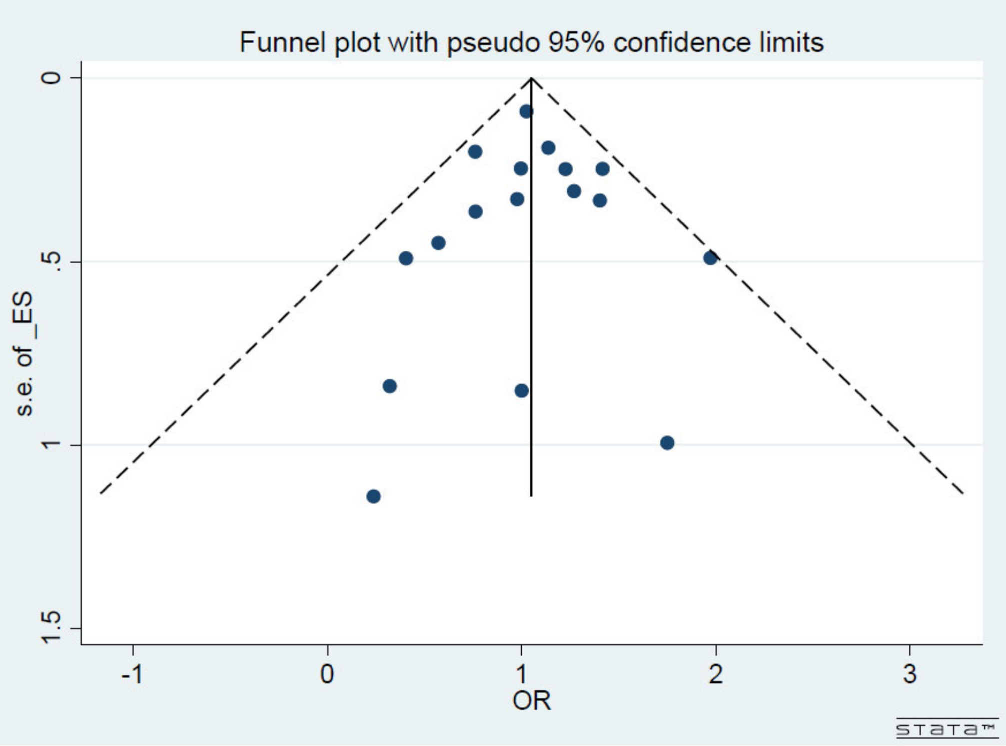

Egger's test for small-study effects: Regress standard normal deviate of intervention effect estimate against its standard error

Number of studies = 26 Root MSE = .7702

Std_Eff	Coef.	Std. Err.	t	P>|t|	[95% Conf. Interval]	
slope	1.190578	.1082619	11.00	0.000	.9671361	1.414019
bias	-.2664412	.2711048	-0.98	0.336	-.825974	.2930915

Test of H0: no small-study effects P = 0.336

© 2021 Teede HJ et al. *JAMA Internal Medicine.*

eFigure 2. Forest Plot of Randomized Controlled Trials and Impact on Gestational Weight Gain

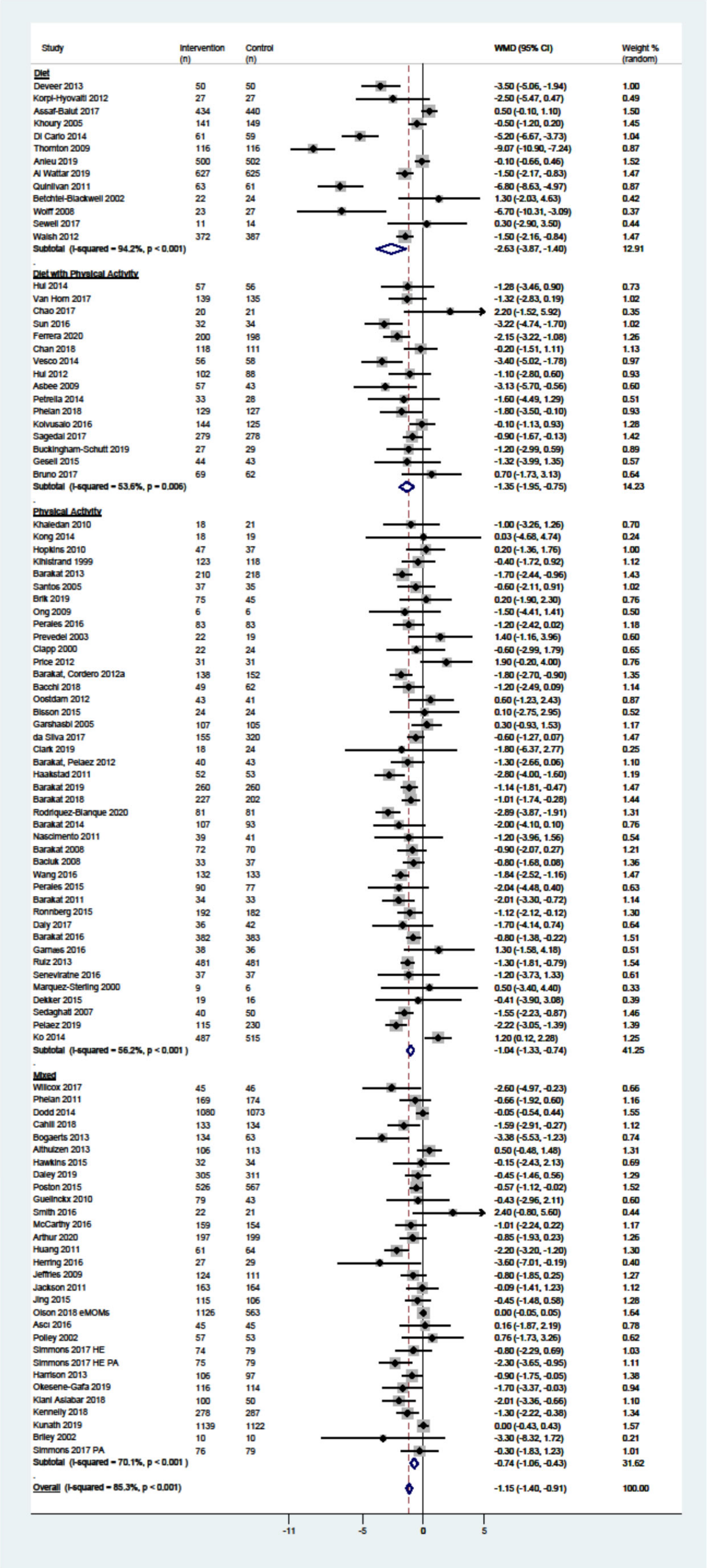

© 2021 Teede HJ et al. *JAMA Internal Medicine.*
